# [Methylhydrazinium]_2_PbCl_4_, a Two-Dimensional Perovskite with Polar and Modulated Phases

**DOI:** 10.1021/acs.inorgchem.2c02206

**Published:** 2022-09-21

**Authors:** Katarzyna Fedoruk, Dawid Drozdowski, Mirosław Maczka, Jan K. Zareba, Dagmara Stefańska, Anna Gagor, Adam Sieradzki

**Affiliations:** † Department of Experimental Physics, Wrocław University of Science and Technology, Wybrzeże Wyspiańskiego 27, 50-370 Wrocław, Poland; ‡ Institute of Low Temperature and Structure Research, Polish Academy of Sciences, ul. Okólna 2, 50-422 Wrocław, Poland; § Advanced Materials Engineering and Modeling Group, Wrocław University of Science and Technology, Wybrzeże Wyspiańskiego 27, 50-370, Wrocław, Poland

## Abstract

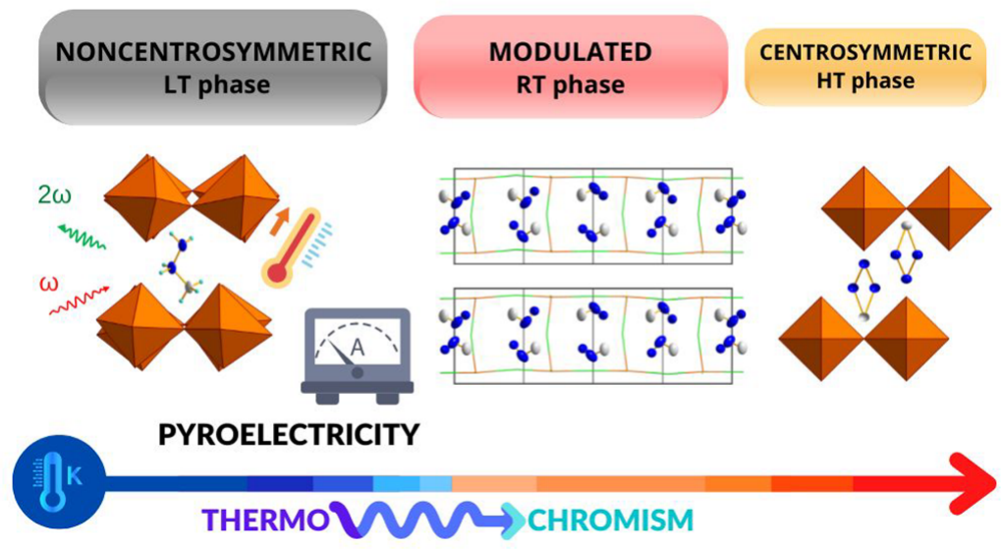

Two-dimensional (2D) lead halide perovskites are a family of materials at the heart of solar cell, light-emitting diode, and photodetector technologies. This perspective leads to a number of synthetic efforts toward materials of this class, including those with prescribed polar architectures. The methylhydrazinium (MHy^+^) cation was recently presumed to have an unusual capacity to generate non-centrosymmetric perovskite phases, despite its intrinsically nonchiral structure. Here, we witness this effect once again in the case of the Ruddlesden–Popper perovskite phase of formula MHy_2_PbCl_4_. MHy_2_PbCl_4_ features three temperature-dependent crystal phases, with two first-order phase transitions at T_1_ = 338.2 K (331.8 K) and T_2_ = 224.0 K (205.2 K) observed in the heating (cooling) modes, respectively. Observed transitions involve a transformation from high-temperature orthorhombic phase **I**, with the centrosymmetric space group *Pmmn*, through the room-temperature modulated phase **II**, with the average structure being isostructural to **I**, to the low-temperature monoclinic phase **III**, with non-centrosymmetric space group *P*2_1_. The intermediate phase **II** is a rare example of a modulated structure in 2D perovskites, with *Pmmn*(00γ)s00 superspace symmetry and modulation vector **q** ≅ 0.25c*. MHy_2_PbCl_4_ beats the previous record of MHy_2_PbBr_4_ in terms of the shortest inorganic interlayer distance in 2D perovskites (8.79 Å at 350 K vs 8.66 Å at 295 K, respectively). The characteristics of phase transitions are explored with differential scanning calorimetry, dielectric, and Raman spectroscopies. The non-centrosymmetry of phase **III** is confirmed with second harmonic generation (SHG) measurements, and polarity is demonstrated by the pyroelectric effect. MHy_2_PbCl_4_ also exhibits thermochromism, with the photoluminescence (PL) color changing from purplish-blue at 80 K to bluish-green at 230 K. The demonstration of polar characteristics for one more member of the methylhydrazinium perovskites settles a debate about whether this approach can present value for the crystal engineering of acentric solids similar to that which was recently adopted by a so-called fluorine substitution effect.

## Abstract 2

The properties of an unknown halide analogue of extant two-dimensional methylhydrazinium perovskites of formula MHy_2_PbCl_4_ were explored. A multitechnique investigation of the structural, polar, dielectric, nonlinear, and linear optical properties of all crystal phases of MHy_2_PbCl_4_ forms the basis of the present contribution.

## 1 Introduction

Considering the unprecedented promise of hybrid organic–inorganic perovskites (HOIPs) for new types of solar cells^1^ and other devices, it is an all-important task to have a more in-depth understanding of the connection between structure and properties in this class of compounds. Perhaps in the photovoltaic context, the most publicity has been gained so far by three-dimensional (3D) perovskites, spearheaded primarily by MAPbI_3_ (MA = methylammonium).^2−5^ 3D perovskites feature a general formula of ABX_3_, where A is an organic or alkali metal cation, B is a divalent metal cation, and X stands for halogen. If A-site cation is organic, it must be small enough to fit into the cubic metal-halide BX_3_^–^ (ReO_3_-type) network. Essentially, only a handful of organic cations are capable of serving as guests within 3D perovskite lattices, and the title methylhydrazinium (CH_3_NH_2_NH_2_^+^, MHy^+^) is one of them. However, there is virtually unrestricted chemical space of 2D HOIPs and low-dimensional (1D, 0D) structures since in these topologies the steric hindrance of the coordination network does not restrict the size of organic cations anymore. This is so for 2D perovskites, which emerge as structurally more diverse alternatives to their 3D counterparts.^6^ Indeed, in addition to changes in organic, metal, and halide components, there is also an opportunity to control the number and thickness of inorganic layers.^7−9^ Critical to the functional properties are the physical implications arising from quantum effects associated with reduced dimensionality. The inorganic layers form slabs of 2D-confined quantum structures, and as a consequence, the formed quantum wells are separated by the organic barriers. The resulting complex electronic structure is sensitive to structural inputs, which can assist in controlling the dielectric and optical properties of these structures.^10−14^ Indeed, quantum and dielectric confinement effects in 2D perovskites increase the effective band gap and the exciton binding energy compared to 3D perovskites.^15^ For instance, the exciton binding energies increase by more than an order of magnitude from ∼10 meV for 3D perovskites to typically >150 meV for conventional 2D perovskites,^15−18^ leading to a radically improved photoluminescence quantum yield (PLQY) for layered analogues.^13,19,20^

It is the innumerable choices of organic components that make possible the tailoring of linear and nonlinear optical (NLO), electrical, and structural properties of perovskites. However, in some cases, simplicity, rather than complexity, yields the most intriguing results. A prime example of that is a small MHy^+^ cation, which in its simplistic structure is unique in that it builds up both 2D and 3D lead halides.^21−26^ In the case of 2D perovskites such as MHy_2_PbBr_4_ and MHy_2_PbI_4_, the small size of this cation translates to a record low separation between inorganic layers (8.91 Å at 300 K and 9.36 Å at 305 K, respectively) and a very small, as for 2D perovskites, estimated exciton binding energy (99.9 and 59.2 meV, respectively).^22,25^ Quite an interesting aspect of using the MHy^+^ cation for construction of perovskites is the apparent guided assembly of non-centrosymmetric perovskite phases. Indeed, the first 3D perovskite reported, with at least one unequivocally acentric phase was MHyPbBr_3_,^21^ soon after followed by the discovery of MHyPbCl_3_ with two acentric crystal phases (monoclinic and orthorhombic), verified by pyrocurrent measurements and capable even for unusual temperature switching of a second-order NLO response of SHG-*low*–SHG-*high* type.^23^ Mixed-halide analogues MHyPbBr_3_Cl_(3-*x*)_ mirror the phase behavior of the MHyPbCl_3_ prototype in that they contain two polar crystal phases, irrespectively of Br content.^24^ MHy_2_PbBr_4_ is ferroelectric at room temperature with orthorhombic *Pmn*2_1_ symmetry.^22^ Among 2D (*n* = 1) MHy^+^-perovskites, only MHy_2_PbI_4_ does not reveal polar properties;^25^ however, it features the unique octahedral tilt system.^6^ The ability of MHy^+^ to direct non-centrosymmetric structures does not work for the 2D and even low-dimensional iodides.^27^ The same applies to other organic cations, such as benzylammonium^28−30^ or cyclohexylammonium,^31,32^ wherein only chloride or bromide analogues adopt polar phases. It may be possibly explained via weakening of hydrogen bonding (HB) strength with decreasing electronegativity of halides.^33^ Thus, the N–H···I HBs are the weakest, and therefore for iodide systems the cations may adopt the most energetically favorable alignment, i.e., without inducing a resultant dipole moment. Polar structure, *Cc*, was also reported for 2D (*n* = 3) BA_2_MHy_2_Pb_3_Br_10_ perovskite (BA = butylammonium).^26^ Accordingly, one sees that there is mounting evidence that the MHy^+^ component promotes the formation of non-centrosymmetric phases, which however requires further experimental verification. Of large comparative value would be to explore the properties of unknown halide analogues of extant methylhydrazinium perovskites, one of which is a 2D analogue of formula MHy_2_PbCl_4_. Indeed, the multitechnique investigation of structural, polar, dielectric, nonlinear, and linear optical properties of all crystal phases of MHy_2_PbCl_4_ forms the content of the present contribution.

## 2 Materials and Methods

### Synthesis

PbCl_2_ (98%, Sigma-Aldrich), methylhydrazine (98%, Sigma-Aldrich), hydrochloric acid (48 wt % in H_2_O, POCH), methyl acetate (99.5%, Sigma-Aldrich), and *N*,*N*-dimethylformamide (DMF, 99.8%) were commercially available and used without further purification. In order to obtain single crystals of MHy_2_PbCl_4_, a reaction mixture containing 15 mmol of methylhydrazine neutralized with HCl (pH = 7), 5 mmol of PbCl_2_, and DMF (about 12 mL) was stirred for an hour until the complete dissolution of PbCl_2_. Then the solution was placed in a glass vial with the lid slightly loosened. This smaller vial was then placed in a second larger glass vial containing methyl acetate with a thoroughly sealed lid. Colorless, plate-like crystals with dimensions of up to 5 mm were harvested after 1 week, filtered from the mother liquid, and dried at RT. A good match of their powder XRD pattern with the calculated one based on the single-crystal data (Figure S1) confirmed the phase purity of the bulk sample. Caution! Methylhydrazine is toxic and must be handled with extreme caution and the appropriate protective gear.

### Differential Scanning Calorimetry (DSC)

Heat capacity was measured using a Mettler Toledo DSC-1 calorimeter with a high resolution of 0.4 μW. Nitrogen was used as a purging gas, and the heating and cooling rate was 5 K min^–1^. The mass of the measured sample was 26.88 mg. The excess heat capacity associated with the PT was calculated by subtracting from the data a baseline representing the system variation in the absence of the PTs.

### Single-Crystal X-ray Diffraction (SCXRD)

SCXRD experiments were carried out using an Xcalibur four-circle diffractometer (Oxford Diffraction) with an Atlas CCD detector and graphite-monochromated Mo Kα radiation. Absorption was corrected by multiscan methods using CrysAlis PRO 1.171.41.93a (Rigaku Oxford Diffraction, 2020). Empirical absorption correction using spherical harmonics, implemented in the SCALE3 ABSPACK scaling algorithm, was applied. For all structures, H atom parameters were constrained. The crystal structures of phases **I** and **III**, and the average structure of phase **II** were solved in Olex2 1.5^34^ using *SHELXT.*(35) Phases **I** and **III** were refined with *SHELXL*.^36^ Refinement of modulated phase **II** was performed using *Jana2020*.^37^ Experimental and refinement details for all phases are summarized in Table S1. Refinement of **I** (*Pmmn* with *a* = 5.7902(1), *b* = 17.5814(6), *c* = 5.8657(1) Å, *V* = 597.13(3) Å^3^, and *Z* = 2) converged to refinement factors *R*_1_ = 0.02, *wR*_2_ = 0.04, and *S* = 1.13. Phase **III** (*P*2_1_ with *a* = 11.6588(5), *b* = 17.0423(6), *c* = 12.7453(6) Å, β = 114.16(1)°, *V* = 2310.65(6) Å^3^ and *Z* = 8) was treated as a two-domain twin. Refinement converged to *R*_1_ = 0.06, *wR*_2_ = 0.17, and *S* = 1.02.

The refinement of modulated phase **II** requires a broader comment. As the satellite reflections appear along c* in ca. one-fourth of the distance between the main reflections (Figure S2), one may treat this phase as a supercell of **I** with a four-fold multiplication of the *c* parameter. However, systematic absences of satellite peaks do not meet the extinction rules known for 3D space groups. Therefore, the (3 + 1) superspace approach was applied. The studied structure was refined in the *Pmmn*(00γ)*s*00 superspace group with modulation vector **q** ≅ 0.25c* (Figure S3).^38^ The refinement of the modulation waves started with the inorganic part. Based on Fourier maps (Figure S4), all of the Pb and Cl atoms were modulated with the assumption of a positional modulation using first-order harmonics. Later, the same approach was applied to the C1 and the terminal N2 atom of MHy^+^, as derived from Fourier maps for MHy^+^ (Figure S5). All of the positional modulation waves were treated as one-dimensional, i.e., propagating along the *a* direction. In the case of the N1 atom, a more accurate refinement was obtained when the occupational modulation was adopted. The occupancy of N1 was modulated with a crenel function with Δ*x*_4_ = 0.5, and the positional modulation functions were described as harmonics in the interval (0,1). Finally, hydrogen atoms were inserted from geometry and refined as riding atoms with *U*_iso_*=* 1.2*U*_iso_ of the maternal atom. The final *R* factors are *R*_1_ = 0.02 (0.09) and *wR*_2_ = 0.03 (0.12) for main reflections (satellites). The displacement of independent Pb and Cl atoms along the *a* direction as a function of the phase of the modulation *t* is shown in Figure S6a. Cl2 demonstrates the highest displacement amplitude of ∼0.20, which is greater than for Cl3 (∼0.15) and for both Pb1 and Cl1 (∼0.07). These displacements reveal variations of Pb–Cl bond lengths (Figure S6b) and Cl–Pb–Cl angles (Figure S6c). It is worth noting here that both from the symmetry relations and from the atomic positions of Pb and Cl atoms the 4c approximant structure of *Pcmn* symmetry is imposed for the II phase (*Pnma* in the standard setting). However, in the 4c superstructure of *Pcmn* symmetry, the amines are disordered over the *m* mirror plane, with two equally occupied positions. Thus, the conventional space group does not resolve the ordering of amines.

### Raman Studies

Temperature-dependent Raman spectra were obtained in the 300–5 cm^–1^ range using a Renishaw InVia Raman spectrometer equipped with a confocal DM 2500 Leica optical microscope, a thermoelectrically cooled CCD as a detector, and an eclipse filter. The excitation was performed using a diode laser operating at 830 nm, and the temperature was controlled using a Linkam cryostat cell. The spectral resolution was 2 cm^–1^.

### Electrical Measurements

Dielectric measurements of the examined samples were carried out using a broadband impedance Novocontrol Alpha analyzer. Electric field-dependent polarization measurements were performed on a single-crystal sample of size 0.4 × 1 × 1 mm^3^. The silver paste was used to ensure good electrical contact. A sinusoidal voltage with an amplitude of 1 V and a frequency in the range of 1 Hz to 1 MHz was applied across the sample. The studies were performed at quasi-static conditions in the temperature range of 150–360 K. The temperature was stabilized using nitrogen gas using the Novocontrol Quattro system. A pyrocurrent measurement was made on a single crystal with silver electrical contact. The crystal was cooled down to 150 K. During cooling, an electric poling field of 200 V/mm was applied. At 150 K, the sample electrodes were shorted for 10 min. Current measurements were performed using a Keithley 6514 electrometer during the heating of the sample from 150 to 300 K with a heating rate of 2 K/min. An aixACCT instrument was used to study the electric field-dependent electric polarization. A periodic triangular signal was used for the measurements. A high voltage was obtained using a Trek 609E6 voltage amplifier. Measurement of the polarization vs field (*P*–*E*) was carried out with a Precision Premier II Ferroelectric tester. The electrodes of conductive silver paste were placed on a single crystal with an area of 0.0226 cm^2^ and a thickness of 345 μm. The measurement was made at a temperature of 180 K. The cooling gas was nitrogen. A maximum voltage of 500 V and frequency of 10^2^ Hz were applied across the sample, and the data were recorded with the Virtual software.

### SHG Studies

Temperature-resolved SHG studies and Kurtz-Perry powder test were performed using a laser system employing a wavelength-tunable Topaz Prime Vis-NIR optical parametric amplifier (OPA) pumped by a Coherent Astrella Ti:sapphire regenerative amplifier providing femtosecond laser pulses (800 nm, 75 fs) at a 1 kHz repetition rate. The output of OPA was set to 1300 nm and was used unfocused. The laser fluence at samples was equal to 0.17 mJ/cm^2^. The single crystals of MHy_2_PbCl_4_ were crushed with a spatula and sieved through an Aldrich mini-sieve set, collecting a microcrystal size fraction of 125–177 μm. Next, size-graded samples were fixed in-between microscope glass slides to form tightly packed layers, sealed, and mounted to the horizontally aligned sample holder. No refractive index matching oil was used. The employed measurement setup operates in the reflection mode. Specifically, the laser beam delivered from OPA was directed onto the sample at 45 deg to its surface. Emission collecting optics consisted of a Ø25.0 mm plano-convex lens of focal length 25.4 mm mounted to the 400 μm 0.22 NA glass optical fiber and was placed along the normal to the sample surface. The distance between the collection lens and the sample was equal to 30 mm. The spectra of the NLO responses were recorded by an Ocean Optics Flame T fiber-coupled CCD spectrograph with a 200 μm entrance slit. Scattered pumping radiation was suppressed with the use of a Thorlabs 750 nm short-pass dielectric filter (FESH0750). Temperature control of the sample was performed using a Linkam LTS420 heating/freezing stage. Temperature stability was equal to 0.1 K. A Kurtz–Perry test was performed by comparing the integral SHG intensity of MHy_2_PbCl_4_ (measured at 160 K) to that of potassium dihydrogen phosphate (KDP) of the same particle size distribution. The same optical setup and laser beam parameters were employed for temperature-resolved studies.

### One-Photon Absorption and Photoluminescence Studies

The RT absorption spectrum of the powdered sample was measured using a Varian Cary 5E UV–vis–NIR spectrophotometer. Emission spectra at various temperatures under 266 nm excitation provided by a diode laser were measured with the Hamamatsu photonic multichannel analyzer PMA-12 equipped with a BT-CCD linear image sensor. The temperature of the single-crystal sample was controlled using a Linkam THMS 600 heating/freezing stage. To record decay times, a femtosecond laser (Coherent Model Libra) was used as an excitation source.

## 3 Results and Discussion

### DSC

**Figure 1:**
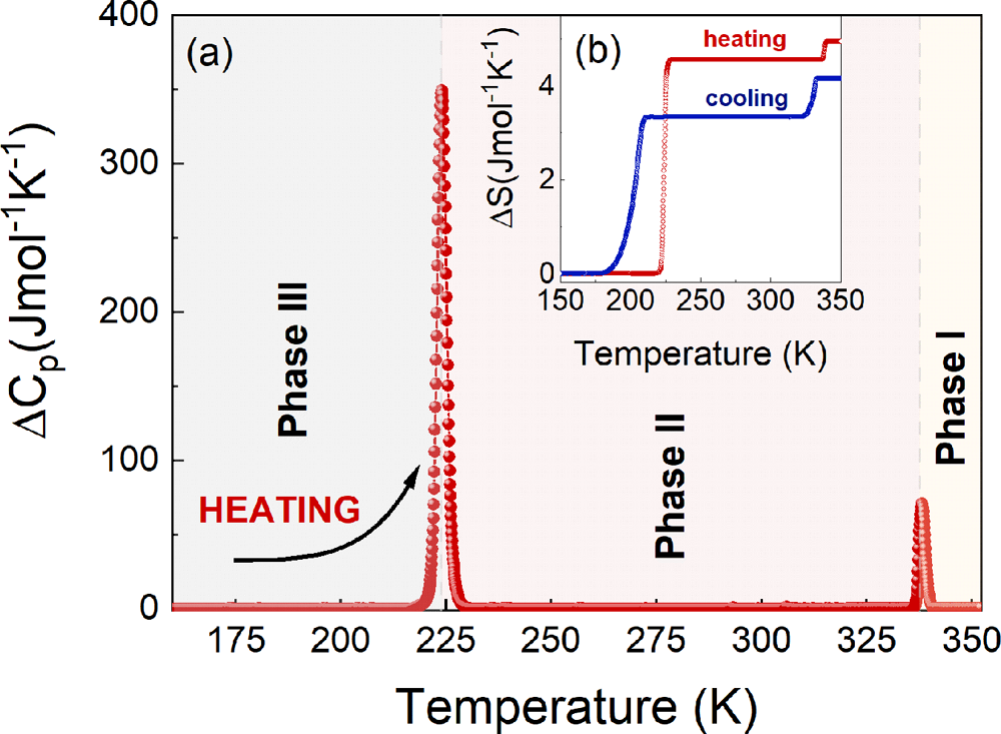
Changes in (a) *C*_*p*_ and (b) *S* related to the PT in the heating (red) and cooling (blue) runs.

The performed calorimetric scans showed two reversible PTs at *T*_1_ = 338.2 K (331.8 K) and *T*_2_ = 224.0 K (205.2 K) visible in the heating (cooling) mode (Figure 1 and Figure S7). The symmetrical, strong peaks seen in Δ*C*_*p*_ plotted as a function of temperature, in combination with the thermal hysteresis between heating and cooling cycles, indicate the first-order type of PTs. This is also confirmed by the accompanying discontinuous change in the entropy (Δ*S*) (see inset in Figure 1b). The changes of entropy (Δ*S*) were estimated to be 0.38 J mol^–1^ K^–1^ (0.82 J mol^–1^ K^–1^) for the PT at *T*_1_ and 4.56 J mol^–1^ K^–1^ (3.33 J mol^–1^ K^–1^) for the PT at *T*_2_ in the heating (cooling) mode, respectively. The symmetry reduction during the transition from HT to modulated phase results in moderate entropy changes. A significantly higher value of Δ*S* at the second PT is associated with symmetry breaking from modulated orthorhombic to monoclinic (see SCXRD studies). Furthermore, the overall change of Δ*S* in MHy_2_PbCl_4_ is much greater than that reported recently for the MHy_2_PbI_4_ (∼2.88 J mol^–1^ K^–1^) and MHy_2_PbBr_4_ (∼1.66 J mol^–1^ K^–1^) analogues.^22,25^ Because this material undergoes a first-order PT, its pressure dependence can be calculated using the indirect Clausius–Clapeyron method and the equation: , where *T*_2_ is the PT temperature, Δ*H*_molar_ (∼1046 J mol^–1^) represents the change in the molar enthalpy, estimated from calorimetric studies, Δ*V*/*V* (∼1.8 × 10^–2^) is the relative volume change at *T*_2_, and *V*_molar_ (∼1.75 × 10^–4^ m^3^ mol^–1^) is the molar unit cell volume (structural data were taken from X-ray diffraction results described below). From the obtained data, the following pressure dependence of *T*_2_ was calculated: K kbar^–1^. The obtained results prove that this compound is an interesting material sensitive to both pressure and temperature changes. The obtained value is one order of magnitude higher than that for [(CH_3_CH_2_CH_2_)_4_N][Mn(N(CN)_2_)_3_],^39^ and two orders of magnitude higher than those for (NH_4_)MF_3_ perovskites (M = Mn, Co, Cd, Mg, and Zn).^40^

### Single-Crystal X-ray Diffraction

MHy_2_PbCl_4_ adopts three temperature-controlled crystal phases. High-temperature (HT) phase **I** possesses the orthorhombic, centrosymmetric *Pmmn* space group. RT phase **II** adopts averaged structure isostructural to **I**; however, as derived from the diffraction pattern (Figure S3), it is a modulated structure with *Pmmn*(00γ)*s*00 superspace symmetry and vector **q** ≅ 0.25c*.^38^ Low-temperature (LT) phase **III** is monoclinic with *P*2_1_ symmetry. Given that this phase is polar, the second-order NLO effects can be expected, e.g., SHG (*vide infra*).

**Figure 2:**
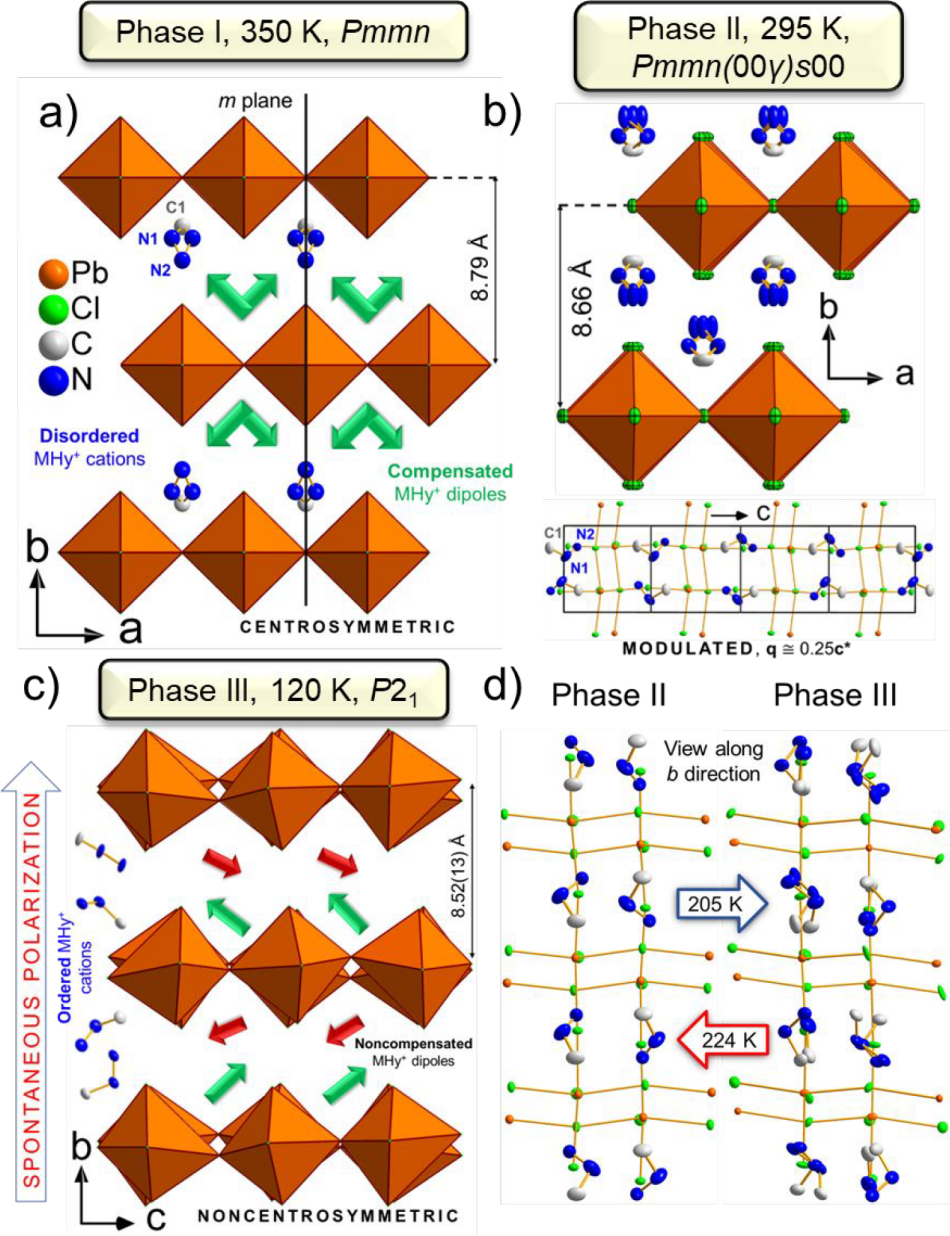
Details of the MHy_2_PbCl_4_ crystal structure in subsequent phases. (a) HT phase **I**, (b) 4-fold approximant of the modulated phase **II**, and (c) LT phase **III**. (d) Comparison of the atomic alignment in **II** and **III** along the [010] direction. The green and red arrows in (a, c) represent locations of the MHy^+^ dipoles.

HT phase **I** is isostructural to the previously reported HT phases of the MHy_2_PbI_4_ and MHy_2_PbBr_4_ 2D analogues.^22,25^ The motif of **I** (Figure 2a) is composed of the corner-sharing [PbCl_6_]^4–^ octahedra forming (010) layers, separated by MHy^+^ cations, which protrude out of the *m* mirror plane. Hence, the N1 atom is split into two equivalent positions with equal probability. The distance between the octahedra layers is equal to 8.79 Å.

The Pb1 coordination sphere consists of the Cl1 and Cl2 atoms (bridging along the *a* and *c* directions, respectively), and the apical Cl3 atom. The individual octahedron is of *C*_2*v*_ symmetry with *mm*2 site symmetry for Pb1, Cl1, and Cl3, and *m*.. for Cl2. Pb–Cl distances vary from 2.74 to 3.12 Å. The difference between the longest and shortest Pb–Cl distances (0.38 Å) is relatively higher than the ones reported for the Br- and I- analogues (0.02 and 0.14 Å for the HT phases of MHy_2_PbI_4_ and MHy_2_PbBr_4_, respectively),^22,25^ pointing to the higher octahedral distortion in the structure described herein. Indeed, both the bond length distortion (Δ_d_) and octahedral angle variance (σ^2^) values (Table 1) are 1.6 × 10^–3^ and 14.4°^2^, which is greater than for MHy_2_PbI_4_ (6.8 × 10^–6^ and 5.6°^2^) and MHy_2_PbBr_4_ (2.1 × 10^–4^ and 11.3°^2^).^22,25^ This distortion results in a decrease of the Pb–Cl–Pb angle along the [100] direction, and its value defined as *Def* = 180° – ∠_Pb–Cl–Pb_ is the highest (*a.d.* = 11.7°) among the MHy_2_PbX_4_ (X = Cl, Br, I) perovskites (where *a.d.* = 9.3° and 10.7° for I and Br, respectively).^22,25^ Observation of the octahedra distortion is strictly connected with intermolecular interactions between the perovskite layers and MHy^+^ cations. However, the interlayer MHy^+^ cation alignment in 2D perovskites diminishes the strength of these interactions when compared to their 3D counterparts. It is noticeable, for instance, in max. σ^2^ values −301°^2^ (314°^2^) for MHyPbBr_3_ (MHyPbCl_3_).^23,24^ Despite relatively weak intermolecular forces, several N–H···Cl hydrogen bonds (HBs) are formed with both N atoms as donors and the Cl1 and Cl3 atoms as acceptors (Table S2).

**Table 1:** Distortion Parameters of the MHy_2_PbCl_4_ Perovskite in the Subsequent Polymorphic Phases

phase	*T* (K)	Pb–Cl–Pb direction	∠Pb–Cl–Pb (deg)	*a.d.* (deg)	*D*_out_ (deg)	max^a^ Δ_d_ × 10^–3^	max^a^ σ^2^ (deg^2^)
**I**	350	[100]	168.27(1)	11.7	0	1.6	14.4
		[001]	180	0	0		
**II**^b^	295	[100]	165.78(1)	14.2	0	1.7	24.7
		[001]	172.34(1)	7.7	0		
**III**	120	[100]	166.1(5)	13.9	6.1	3.4	61.4
		[001]^c^	160.9(5)	2.1	19.1		

a Maximum distortion parameters are provided due to the existence of four inequivalent [PbCl_6_]^4–^ octahedra in phase **III**.

b Distortion parameters are given for modulated phase **II**.

c The direction maintained from the axis setting of phases **I** and **II**. *a.d.* = 180 – ∠_Pb–Cl–Pb_ along [100] and [001] directions; *D*_out_ = 180 – ∠_Pb–Cl–Pb_ along [010]; Δ_d_ – bond length distortion; σ^2^ – octahedral angle variance.^41^

Lowering the temperature induces **I** → **II** PT. The effect of the unit-cell contraction as the temperature decreases leads to the reduction of the interlayer distance to approximately 8.66 Å. While the average structure of **II** is isostructural to **I**, satellite diffraction peaks along the c* direction appear in the diffraction pattern (Figure S2). As derived from the systematic absences (see caption to Figure S3), the (3 + 1)-dimensional superspace group of **II** is *Pmmn*(00γ)*s*00 with the modulation vector **q** ≅ 0.25c*. The 4-fold approximant of **II** is presented in Figure 2b. As compared to **I**, the atoms of the inorganic part slightly lean out of the mirror plane toward the [100] direction (Figure S8). This behavior brings consequences to the distortions of the perovskite layers. To start with, the change of the Pb–Cl–Pb angle along [001] appears with *a.d.* = 7.7°. Simultaneously, the maximum *a.d.* alongside [100] increases to 14.2°. Additionally, both Δ_d_ and σ^2^ values increase to 1.73 × 10^–3^ and 24.7°^2^, respectively (Table 1, Figure S9a,b). In the case of the organic part, the MHy^+^ cation in **II** is ordered, and its distribution in the structure is governed by the additional translation component of the superspace group (intrinsic phase shift of 1/2) associated with the 2-fold axis (lower part of Figure 2b). This new configuration of amines is associated with the appearance of N–H···N and N–H···Cl HBs. However, the juxtaposition of distances between N atoms of neighboring MHy^+^ as a function of the phase of the modulation *t* (Figure S9c) indicates that in **II** there are NH_2_ groups that are not involved in hydrogen bonding. There is no N–H···Cl or N–H···N stable HBs network along the [001] direction (Figure S10a).

With further temperature lowering, the shrinking of interatomic distances strengthens the interactions between inorganic and organic constituents and eventually leads to the formation of a new phase with a stable configuration of HBs. Indeed, such a phase appears at 205.2 K (224.0 K) on cooling (heating). The LT phase **III** is monoclinic with a *P*2_1_ polar space group and β = 114.16(1)° with an asymmetric unit consisting of 4 lead cations, 16 chloride anions, and 8 ordered MHy^+^ cations (Figure S11). The main details of **III** are shown in Figure 2c and compared to modulated phase **II** in Figure 2d. Unlike the HT and RT phases, in **III**, certain MHy^+^ cations are spontaneously rotated. From the viewpoint of polar characteristics, MHy^+^ dipoles are no longer compensated. Instead, spontaneous polarization along the [010] direction occurs. Furthermore, due to the reduced interlayer gap of 8.52(13) Å, numerous HBs are formed (Table S3). One may distinguish two kinds of HBs. The first kind is N–H···Cl with N atoms acting as donors and Cl atoms (mainly apical) as acceptors. Contrarily to **II**, these HBs create the network alongside both directions of propagation of the octahedra (Figure S10b). The second kind of HBs is N–H···N, where both N atoms may serve as donors and acceptors for the hydrogen atoms. As the **II → III** PT is associated with symmetry reduction, all atoms in the unit cell adopt a general *C*_1_ site. The synergistic effect of strengthened intermolecular interactions and the symmetry reduction remarkably influences the shape of the perovskite layers. Besides the in-plane Pb–Cl–Pb angle deformation (max. *a.d.* = 13.9°), the out-of-plane octahedra tilting is observed with the *D*_out_ value of 19.1°. At the same time, the octahedra distortion parameters significantly increase (Δ_d_ = 3.4 × 10^–3^ and σ^2^ = 61.4°^2^, Table 1). Lastly, the **II → III** PT provokes a step change in lattice parameters (Figure S12a) and an enlargement of a unit cell volume (Figure S12b).

In this paragraph, a wider comment is provided on the interlayer distances in the reported compound. As indicated above, the gap between perovskite layers decreases in the order **I → II → III** (8.79, 8.66, and 8.52 Å, respectively). It is worth noting that such small values were not recorded before in the 2D lead chloride perovskites with [PbCl_6_]^4–^ monolayers (Table S4). At the same time, MHy^+^ is the smallest organic cation among the listed ones. This observation agrees with the hypothesis provided in our previous paper concerning the MHy_2_PbBr_4_ analogue,^21^ that the MHy^+^ cation is a key component developing 2D hybrid perovskites with a record-breaking low layer separation.

### Raman Studies

**Figure 3:**
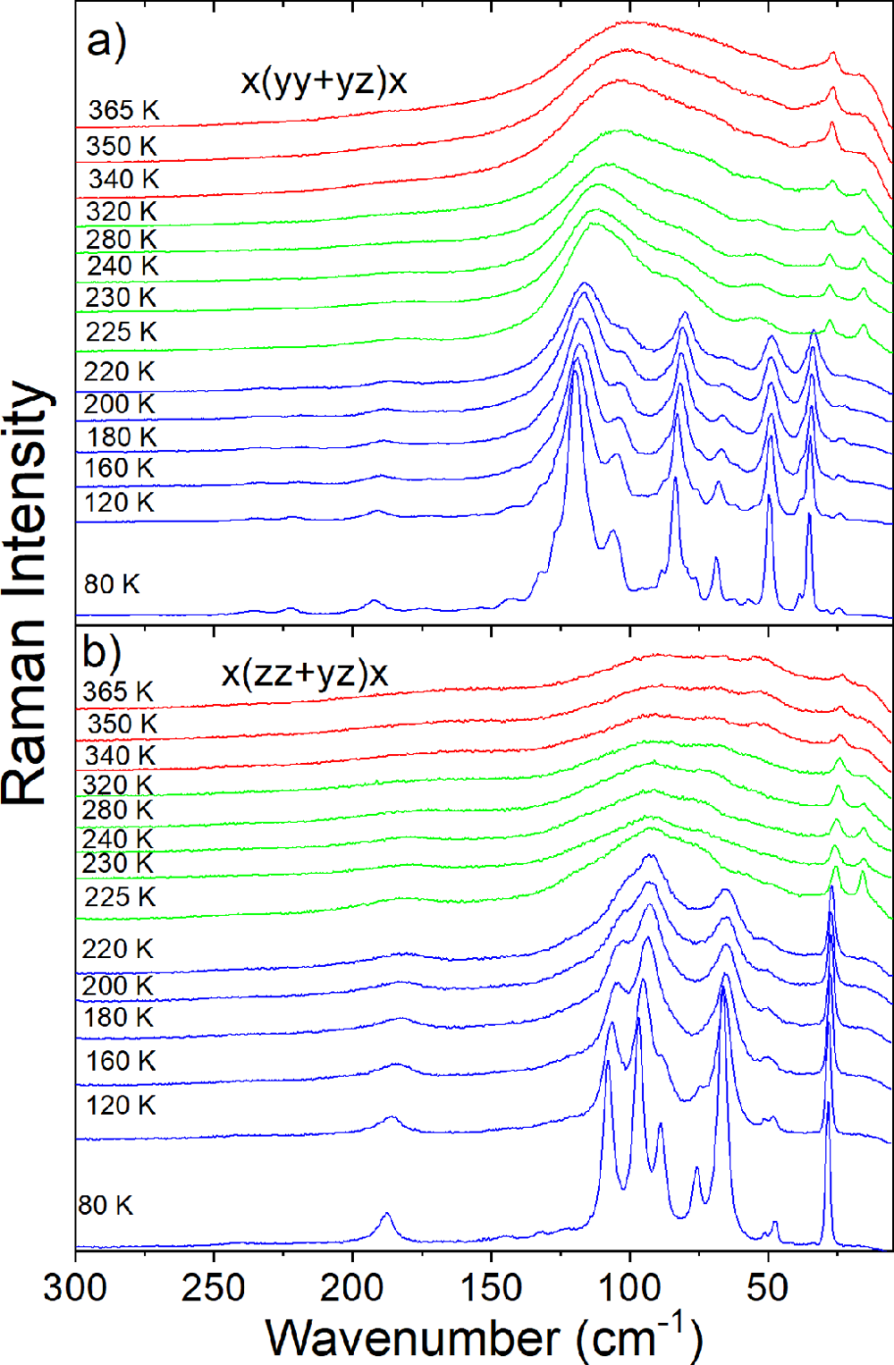
Temperature-dependent Raman spectra of MHy_2_PbCl_4_ crystals in a heating run for (a) *x*(*yy* + *yz*)*x* and (b) *x*(*zz* + *yz*)*x* polarization. Red, green, and blue colors correspond to the *Pmmn* phase **I**, modulated phase **II**, and *P*2_1_ phase **III**, respectively.

In order to obtain further insight into the mechanism of the PTs and lattice dynamics, temperature-dependent Raman studies were performed on MHy_2_PbCl_4_ single crystals in the low-wavenumber range of 300–5 cm^–1^ (Figure 3). The observed Raman modes are tabulated in Table S5. According to previous studies of layered perovskites, PEA_2_PbBr_4_ and BA_2_PbBr_4_ (PEA = phenylethylammonium; BA = butylammonium) Raman spectra in the low-wavenumber range consist of five ranges. The lowest wavenumber narrow bands, which do not exhibit any splitting at LT, correspond to the octahedra rocking/twisting that could be alternatively assigned to PbBr_6_ librational modes.^42^ These modes were observed at near 27 and 22 cm^–1^ for PEA_2_PbBr_4_ and BA_2_PbBr_4_, respectively.^42^ Raman spectra of MHy_2_PbCl_4_ show such narrow modes at 24 and 29 cm^–1^ (values at 80 K, Table S5). The corresponding modes of MHy_2_PbBr_4_ and MHy_2_PbI_4_ were observed at 23 and 17 cm^–1^, respectively.^25,43^ As can be seen, these modes shift weakly to higher wavenumbers with the decreasing size of the halide anion. The most intense Raman bands (weak bands), observed for PEA_2_PbBr_4_ and BA_2_PbBr_4_ in the 80–35 cm^–1^ range (140–83 cm^–1^ range), were attributed to Pb–Br bond bending modes (Pb–Br bond stretching modes).^25^ These broad bands at RT exhibited splitting into many components at LT.^25^ For the HT phase of MHy_2_PbCl_4_, Pb–Cl bending and stretching modes are located in the 105–54 and 186–164 cm^–1^, respectively (Table S5). It is worth noting that these bands exhibit a very pronounced shift to lower wavenumbers with the increasing size of the halide anions, i.e., to 47–45 and 137–116 cm^–1^ for MHy_2_PbBr_4_ and 34–27 and 120–97 cm^–1^ for MHy_2_PbI_4_.^25,43^

According to the X-ray diffraction studies, MHy^+^ cations are disordered in phase **I** and ordered in phase **II**. One would expect, therefore, to observe a significant narrowing of bands at the PT temperature. As can be noticed, Raman bands exhibit a rather weak narrowing at *T*_1_ (see Figure 3). Interestingly, very similar behavior was also reported for MHy_2_PbBr_4._^43^ Thus, in both analogues, the dynamics of MHy^+^ cations does not exhibit a sudden change at the PT temperature. Nevertheless, the PT leads to some changes in the intensity of Raman modes. For instance, a new L(PbCl_6_) band appears at 16 cm^–1^, and in the *x*(*zz*+*yz*)*x* polarization intensity of the 54 cm^–1^ band drastically decreases (Figure 3). However, the observed changes at *T*_1_ are weak, providing a spectroscopic argument for the significant crystallographic similarity of phases **I** and **II**.

On further lowering of temperature, lattice modes exhibit very pronounced changes when the temperature decreases from 225 to 220 K (Figure 3). First, Raman modes exhibit significant shifts and split into many components. Second, Raman bands decrease fwhm, and they become very narrow at 80 K. All of these changes point to further slowing down of the MHy^+^ cation dynamics and a significant decrease in crystal symmetry, associated with the change of distortion of PbCl_6_ octahedra and appearance of out-of-plane tilting. Note that the number of Raman bands observed at 80 K is much larger for MHy_2_PbCl_4_ (24 in the *x*(*yy*+*yz*)*x* polarization), compared to MHy_2_PbBr_4_ (nine bands).^41^ This observation gives strong evidence that the crystal structure of the lowest temperature phase of MHy_2_PbCl_4_ features significantly lower symmetry than the crystal structure of its bromide analogue. This conclusion is consistent with X-ray diffraction data that revealed *Pmn*2_1_ and *P*2_1_ symmetry for the lowest temperature phases of MHy_2_PbBr_4_ and MHy_2_PbCl_4_, respectively.

### Electrical Measurements

**Figure 4:**
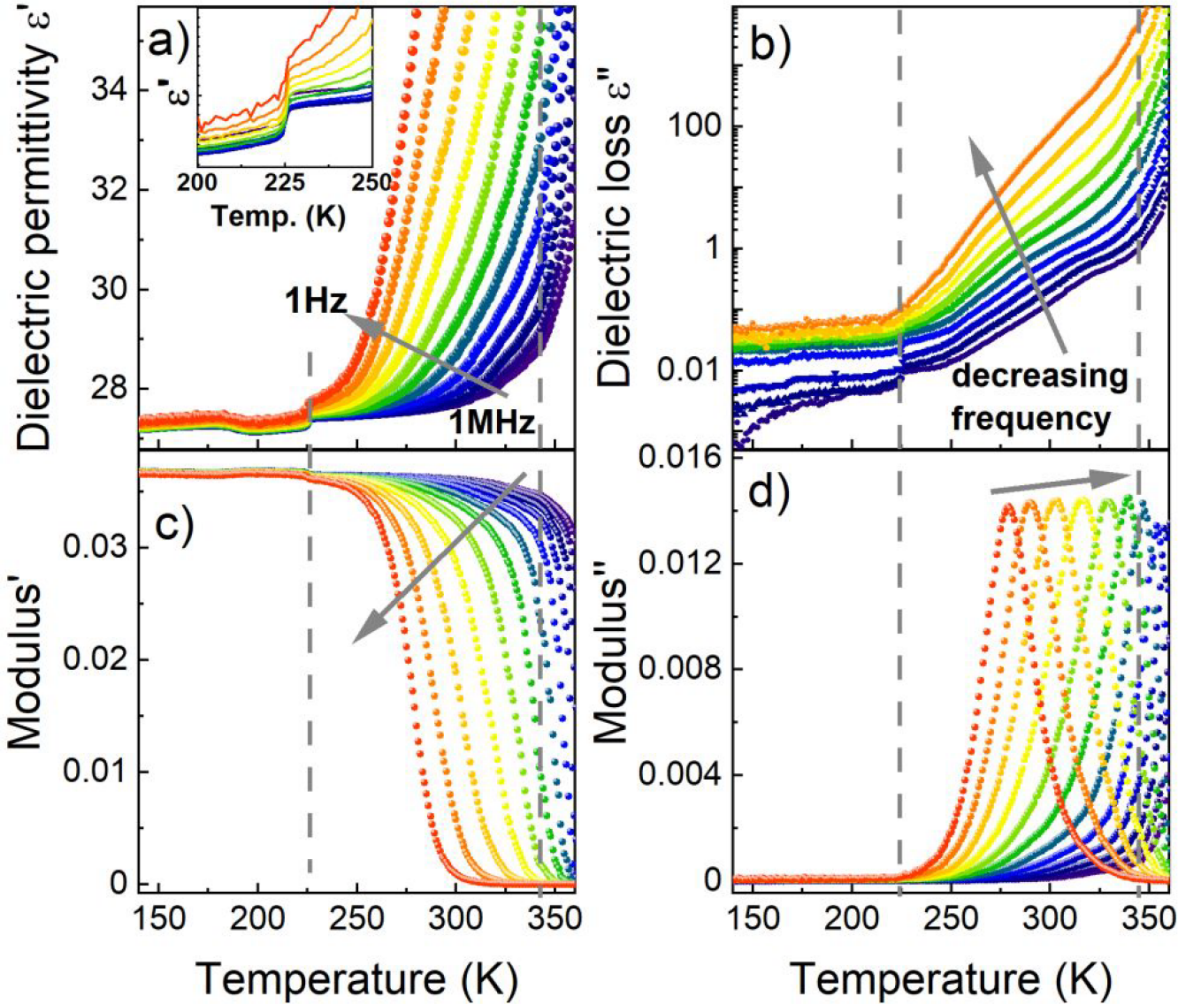
Temperature dependence of the (a) dielectric permittivity, (b) dielectric loss, (c) real M′, and (d) imaginary M″ components of electric modulus spectra as a function of the temperature of the MHy_2_PbCl_4_ single crystal along the [010] direction measured on heating. The representative curves are plotted in frequency decades between 1 Hz and 1 MHz. Dashed lines correspond to the structural PT temperatures. The changes in dielectric permittivity for the area near the PT are presented in the inset in a.

A broadband dielectric spectroscopy measurement was carried out to investigate the dipolar relaxation arising from the reorientation motions of molecular dipoles and conduction arising from the translational motions of electric charges (ions, electrons). Temperature-dependent complex dielectric permittivity ε* (ε* = ε′ – iε″, where ε′ is the dielectric permittivity, and ε″ is the dielectric loss, respectively) is presented in Figure 4a,b. A slight, step-like anomaly of ε′ can be observed at ∼224 K on heating, related to the structural PT from phase **II** to phase **III**. However, above the observed PT, frequency dispersion can be noticed for ε*, which is probably associated with ionic/electrical conductivity processes. To reduce the contribution of the conductivity component at higher temperatures, the modulus representation (M* = 1/ε*) was used (Figure 4c,d).^44^ The bell-shaped spectra of M″ and the step-like curves of M′ shift toward HTs with increasing frequency, which implies the presence of conduction and dielectric relaxation processes. The visible decrease in the value of the M″ coincides with the temperature of the PT from phase **I** to phase **II**. In order to investigate this behavior, the dependence of ε* and M* as a function of frequency in the temperature range of 140–360 K was performed (Figure S13). The visible bell-shaped spectra of M″ allow the activation energy to be determined. The data were parametrized in the vicinity of the peak maximum with the use of the single Havriliak–Negami function. It was noticed that in the studied temperature range, the relaxation times (τ) exhibit linear tendencies as a function of the inverse temperature (1000/*T*). Therefore, the relaxation times can be modeled using the Arrhenius relation:
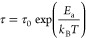
where τ_0_, *k*_B_, and *E*_a_ are relaxation times at the high temperature limit, Boltzmann constant, and activation energy, respectively. Based on this estimation, the *E*_a_ in phase **I** is 1.8 eV and in phase **II** is 0.7 eV (Figure S14). Taking into account the X-ray diffraction data, the mechanism of the observed dielectric relaxation process in MHy_2_PbCl_4_ can be related to the new configuration of MHy^+^ and/or ionic conductivity. The obtained values of *E*_a_ are slightly higher than those reported for related compounds such as [triethylpropylammonium]PbI_3_ (0.66 eV in the HT phase and 1.09 eV in the LT phase),^45^ MHy_2_PbI_4_ (0.48, 0.80, and 0.70 eV from the HT phase to the LT phase, respectively).^25^

**Figure 5:**
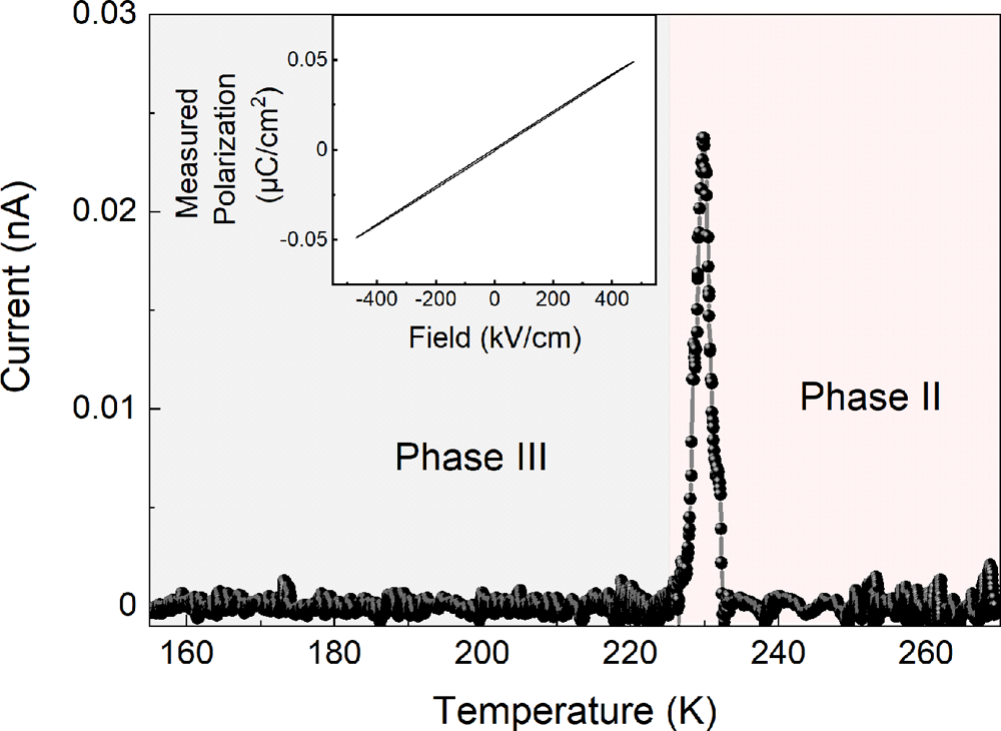
Temperature dependence of the pyroelectric current of a single crystal after poling in the DC electric field. Inset shows measured polarization as a function of the applied electric field at a temperature of 180 K.

The temperature-dependent pyrocurrent of the MHy_2_PbCl_4_ single crystal measured along the [010] direction is presented in Figure 5. We observe a sudden increase in the value of the pyrocurrent during the heating of the sample at the PT temperature of about 224 K, confirming the material’s polar properties. Despite repeated trials, we could not obtain a *P*–*E* hysteresis loop for MHy_2_PbCl_4_, which indicates that it is a pyroelectric material. A similar case was observed for the related 3D compound MHyPbCl_3_.^23^

### Temperature-Resolved SHG Studies

Despite the fact that 2D HOIPs have been around for some time, their diverse NLO properties have gained significant interest only in the past few years. As is the case with the majority of the 2D materials, the NLO properties of 2D HOIPs are associated with their quantum-well structure; it is influenced by the quantum and dielectric confinement introduced by inorganic and organic layers, respectively.^46^ Such systems typically possess strong two- and multiphoton excited PL due to large nonlinear absorption cross sections and luminescence quantum yields.^47^ This is broadly true for the majority of 2D perovskites, particularly those comprising bromine, iodine, or a mixture of those at the so-called X-site.^48^ The SHG phenomenon can also benefit from confinement effects but, on the other hand, has a strict non-centrosymmetry requirement imposed by the χ^(2)^ tensor, making this process possible only in acentric crystalline materials. For this reason, the SHG-active 2D HOIPs are not really common; the most notable examples include Ruddlesden–Popper phases such as (PEA)_2_(MA)_*n*−1_Pb_*n*_I_3*n*+1_ (PEA = phenylethylammonium),^49^ (BA)_2_PbCl_4_ (BA = benzylammonium)^50^ (CH_3_(CH_2_)_3_NH_3_)_2_(CH_3_NH_3_)_*n*−1_Pb_*n*_I_3*n*+1_ (*n* = 1, 2, 3, 4, ∞)^9^ as well as MHy_2_PbBr_4_,^22^ a bromine analogue of the title compound. For this reason, we set out to investigate SHG properties of all crystal phases of MHy_2_PbCl_4_, in particular, to check how they compare to those of MHy_2_PbBr_4_.

**Figure 6:**
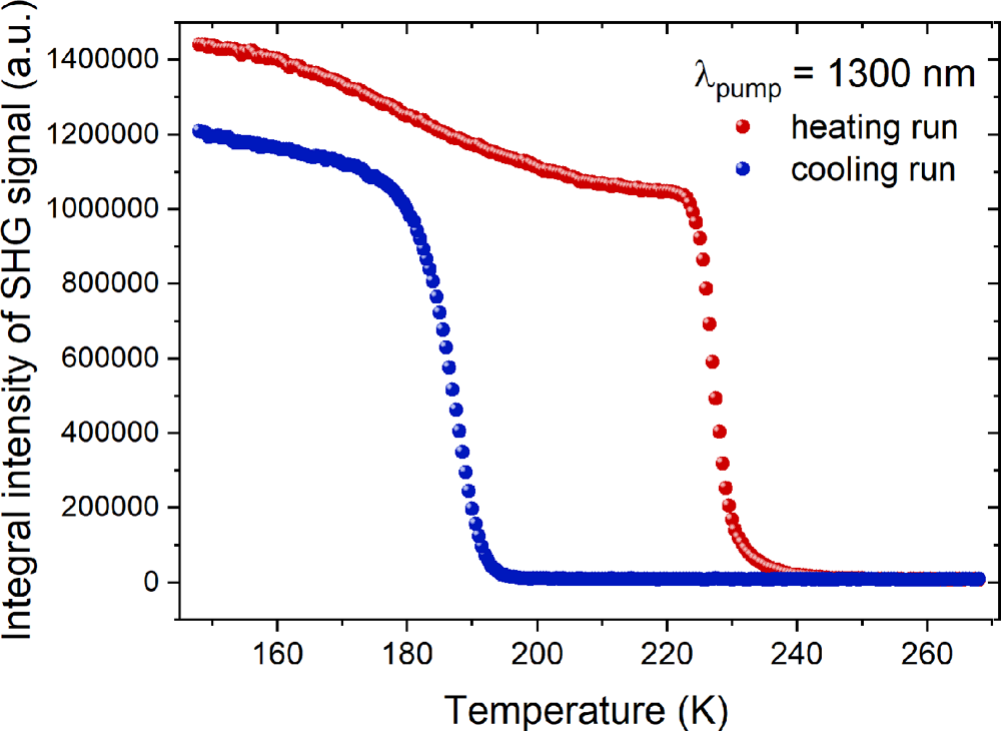
Plots of integral intensities of the SHG signal of MHy_2_PbCl_4_ for heating (red circles) and cooling (blue circles) runs.

A temperature-resolved SHG study of MHy_2_PbCl_4_ was performed with the use of 1300 nm femtosecond laser pumping; this wavelength was chosen to enable comparisons with extant methylhydrazinum-based perovskites. First, we will discuss TR-SHG results for the RT phase **II** and LT phase **III**. Figure 6 presents integral areas of SHG signals (λ_SHG_ = 650 nm) plotted as a function of temperature for heating (148–268 K) and cooling run (268–148 K), while experimental spectra of NLO responses are displayed in Figure S15. These data show that MHy_2_PbCl_4_ is clearly SHG-active in its LT phase **III**, while PT to phase **II** (at ca. 223 K upon heating) results in a complete loss of SHG activity. Upon cooling at ca. 193 K, SHG starts to be restored due to a return to the non-centrosymmetric phase **III**. PT between phases **II** and **III** is therefore reversible, as well as features broad hysteresis, which confirms its first-order character. At this point, it should be noted that the LT phase **III** of MHy_2_PbBr_4_ is also non-centrosymmetric.^22^

In a separate experiment, we checked whether the HT phase **I** is SHG-active. To this end, we performed a temperature scan (300–355 K) using the same irradiation conditions and registered spectra of nonlinear emissions. As seen in Figure S16, spectra in this temperature range do not show any contribution of SHG at 650 nm, but only a third-harmonic generation (THG) signal at 433 nm, which is present for each phase regardless of its symmetry (compare with Figure S15). Accordingly, the centrosymmetric character of phase **I**, inferred from crystallographic data, is confirmed by these results.

In order to estimate the efficiency of the generation of the second harmonic of radiation by phase **III**, we performed the Kurtz–Perry powder test in which we compared the SHG emission of MHy_2_PbCl_4_ cooled down to 160 K with that of KDP of the same particle size, but measured at RT. It turns out that the relative efficiency of SHG is about 0.21 times that of KDP at 1300 nm. This value is two times higher than that found for MHy_2_PbBr_4_.^22^

From the viewpoint of NLO properties, the title compound is similar to its bromine analogue, MHy_2_PbBr_4_. The fundamental common feature is that both analogues possess three temperature-dependent crystal phases, among which only the LT phase **III** is SHG-active. In fact, in the case of MHy_2_PbCl_4_, the THG is present in addition to SHG. By contrast, phase **III** of MHy_2_PbBr_4_ showed multiphoton-excited luminescence of mixed 3- and 4-photon absorption origin in addition to SHG and THG emissions. This difference can be ascribed to the wide optical bandgap of MHy_2_PbCl_4_ (3.75 eV, see optical properties section) which effectively shifts nonlinear absorption resonances to shorter wavelengths.

All in all, the collected results demonstrate one more example of HOIP that contains MHy^+^ cation and whose at least one crystal phase is SHG-active (acentric). While MHy_2_PbCl_4_ in its crystal architecture and phase behavior mirrors the MHy_2_PbBr_4_ analogue in many respects, it should be pointed out that MHy-containing 3D perovskites, MHyPbCl_3_, MHyPbBr_3_, and halide-mixed MHyPbBr_3_Cl_(3-*x*)_ analogues, possess acentric phases as well.^21,23,24^ In fact, these materials are the only representatives of 3D perovskites that reveal clear polar order. The apparent accumulation of examples of MHy-containing perovskites which are non-centrosymmetric raises the question of whether the MHy^+^ cation—a nonchiral molecule—facilitates crystallization of acentric perovskite phases. If there is indeed something to that, the MHy^+^ component could play a similar role in materials science as recently adopted by amine fluorination,^51−54^ and as such, could be seen as a useful tool for inducing non-centrosymmetry-driven properties, e.g., piezoelectricity or ferroelectricity.

### Photoluminescence

The diffuse reflectance spectrum of MHy_2_PbCl_4_ shows a narrow band positioned at 344 nm (3.60 eV) (Figure S17), which can be assigned to the excitonic absorption. Based on this result, the energy band gap (*E*_g_) of the investigated perovskite was calculated using the Kubelka–Munk equation:^55^
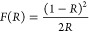
where *R* indicates reflectance. The estimated *E*_g_ value of MHy_2_PbCl_4_ is 3.75 eV (Figure S18); i.e., it is significantly larger than the *E*_g_ of the bromide (MHy_2_PbBr_4_ ≈ 3.02 eV) and iodide (MHy_2_PbI_4_ ≈ 2.20 eV) analogues.^22,25^ The increase of the energy band gap when larger I^–^ are replaced by smaller Br^–^ or Cl^–^ ions can be attributed to the lower electronegativity of the smaller halogen atoms.^20^ The band gap of MHy_2_PbCl_4_ is also larger than the band gap of its 3D MHyPbCl_3_ analogue, for which *E*_g_ = 3.4 eV.^23^ The same behavior, attributed to quantum confinement effects, was also observed for other lead halides.^56^ It is worth adding that the energy band gap and the excitonic absorption of 2D lead halide perovskites with formula A_2_PbX_4_ also become larger with a decrease in the interlayer distance.^22,25,29,57,58^ Thus, we showed that MHy_2_PbI_4_ and MHy_2_PbBr_4_ perovskites exhibit exceptionally small band gaps and the most red-shifted excitonic absorption among all known A_2_PbI_4_ and A_2_PbBr_4_ analogues.^22,25^ The same applies to MHy_2_PbCl_4_, wherein the band gap is smaller (excitonic absorption red-shifted) than the band gaps (excitonic absorption) of A_2_PbCl_4_ (100) perovskites containing large alkylammonium or aromatic cations.^29,59,60^

**Figure 7:**
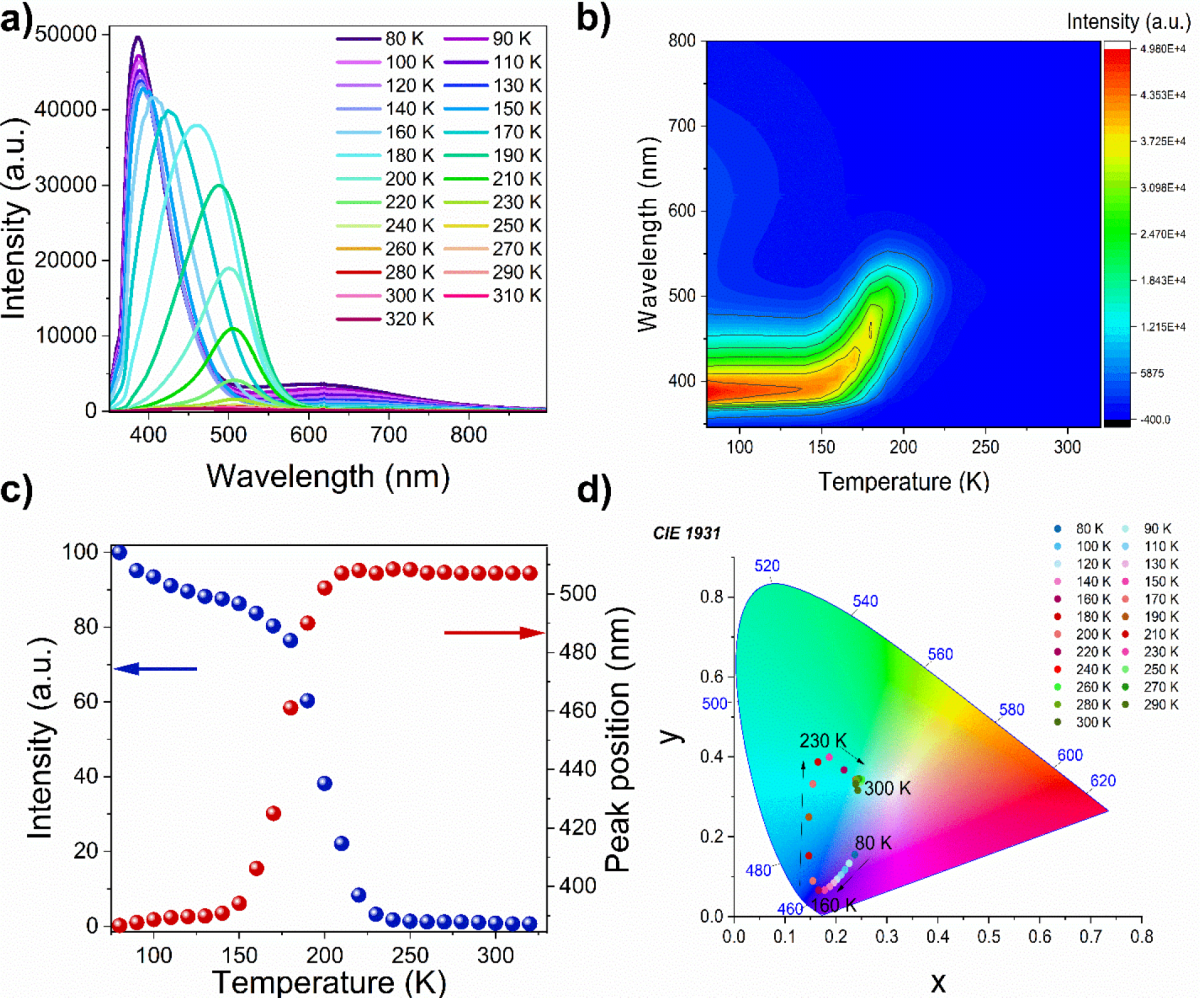
(a) Temperature-dependent emission spectra of MHy_2_PbCl_4_ measured every 10 K from 80 to 320 K, (b) the temperature dependence of band intensity (contour map), (c) the emission intensity as a function of temperature (left side) and the shift of the band center position with temperature, (d) CIE coordinates of MHy_2_PbCl_4_ at various temperatures.

The PL spectrum recorded at 80 K presents a relatively broad band (full width at half-maximum, fwhm = 59.5 nm) centered at 387 nm and a very broad band (fwhm = 250 nm) located at 609 nm (Figure 7a). A significant fwhm and Stokes shift of the first band excludes its assignment to a free exciton (FE) recombination and suggests that it can be attributed to a bound exciton (BE) states or defects corresponding to imperfect stacking of layers (crumpled excitons, CE).^22,24,29,61,62^ Previous studies of layered perovskites often revealed the presence of broad red-shifted bands, which were attributed to the self-trapped excitons (STEs) in the radiative centers.^63,64^ Typically, the PL attributed to STE is observed below 150 K and was reported for some other two-dimensional lead chlorides with a strongly distorted framework as well as 3D MHyPbCl_3_.^19,23,59^ We assign, therefore, the broad band of MHy_2_PbCl_4_ near 609 nm to STEs. The emission decay times of the purplish-blue band (λ_em_ = 387 nm) and the red one (λ_em_= 609 nm) recorded using a 266 nm line generated by the femtosecond laser are nonexponential with a time of τ_1_ = 2.2 ns, τ_2_ = 5.55 ns, and τ_1_ = 0.69 ns; τ_2_ = 3.0 ns, respectively (Figure S19).

As can be seen in Figure 7a–c, the intensity of the band located in the red region rapidly decreases on heating and becomes almost invisible above 130 K. The intensity of the purplish-blue band also decreases on heating, and this band exhibits significant broadening above 150 K. Furthermore, the position of the band maximum shows a pronounced red shift from 394 nm at 150 K to 507 nm at 210 K (Figure 7a–c). This pronounced broadening and the red shift of the PL above 150 nm can be most likely attributed to the light-induced permanent defects.

Based on temperature-dependent emission spectra, the activation energy for the thermal quenching was calculated as follows:
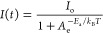
where, *I*_0_, *E*_a_, and *k*_B_ denote the emission intensity at LT, the activation energy, and the Boltzmann constant, respectively. After simple modification, the equation can be rewritten as
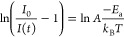


Figure S20 presents ln(*I*_0_/*I*(*T*) – 1) in a linear function of 1/*k*_B_*T*. The activation energy for the thermal quenching of the MHy_2_PbCl_4_ emission equals 366 meV, which is consistent with *E*_a_ values reported for many other A_2_PbX_4_ compounds^19,65^ but is significantly larger than *E*_a_ reported for FE PL in MHy_2_PbI_4_ (59.2 meV) and MHy_2_PbBr_4_ (99.9 meV).^22,25^

The natural consequence of the broadening of the emission band and changing its position with temperature is the shift of the color rendering index CIE (Figure 7d). From 80 to 160 K sample exhibits purplish-blue emission but then the chromaticity coordinates (x, y) change more significantly and the color of the emission tunes rapidly with temperature. As a consequence, the sample glows bluish-green at 230 K.

## 4 Conclusion

HOIPs not only engender emerging technological interest but serve as a unique platform for fundamental studies of structure–property–function relationships. There are organic building units that facilitate the formation of specific types of structures, which can be intentionally employed to increase the likelihood of the generation of desired molecular arrangements. A growing body of evidence suggests that such a structure-directing role in HOIPs may be performed by the methylhydrazinium (MHy^+^) cation in two aspects: (i) preferred formation of non-centrosymmetric perovskite phases; and (ii) self-assembly of 2D HOIPs with an unusually low separation between inorganic layers. With these working hypotheses in mind, in this work, we sought to employ MHy^+^ for the construction of a missing member of the methylhydrazinium 2D perovskites, MHy_2_PbCl_4_.

Indeed, crystallographic results MHy_2_PbCl_4_ show that LT phase **III** is non-centrosymetric (*P*2_1_ space group), while RT phase **II**, featuring the modulated phase of *Pmmn*(00γ)s00 superspace symmetry and HT phase **I** (orthorhombic, *Pmmn*) are both centrosymmetric. The acentric setting of phase **III** is corroborated by SHG studies as well as by the pyroelectric effect, showing the decay of spontaneous polarization along the [010] crystallographic direction upon PT. However, polarization could not be switched by the application of an external electric field; hence, MHy_2_PbCl_4_ cannot be considered ferroelectric.

Structural studies also demonstrate that MHy_2_PbCl_4_ establishes a new record for the short distance between inorganic layers in 2D HOIPs (8.79 Å at 350 K), breaking the previous one held by a bromine analog of the formula MHy_2_PbBr_4_. Nevertheless, in this case, the short interlayer distance does not translate to an unusually low exciton binding energy as was noted for bromine and iodine analogs. On the contrary, the value of exciton binding energy, estimated based on the activation energy for the thermal quenching equals 366 meV and is in the range typical for 2D perovskites. Further optical studies revealed that MHy_2_PbCl_4_ on heating displays a marked broadening and redshift of the higher energy band, which can most likely be attributed to light-induced permanent defects. The estimated *E*_g_ value of MHy_2_PbCl_4_ is 3.75 eV, which is significantly larger than those of the bromide and iodide analogues.

All in all, MHy_2_PbCl_4_ constitutes a plain example of MHy^+^-directed formation of a non-centrosymmetric phase in 2D perovskite combined with the structural effect of an unusually low separation between inorganic layers. The guided formation of acentric phases by MHy^+^ resembles that observed for ferroelectric materials comprising fluorine-substituted organic ligands. MHy_2_PbCl_4_ features a thermally responsive lattice, as revealed by dielectric and Raman measurements. By using the Clausius–Clapeyron relation, it was demonstrated that MHy_2_PbCl_4_ is not only responsive to temperature, but its structure should also display strong structural responsiveness to pressure stimuli, warranting further studies of pressure-dependent physical properties.

## Supporting Information Available

Figures: DSC traces, projections of crystal structures, dielectric curves, energy activation, SHG spectra, THG spectra, diffuse reflectance, emission spectra. Tables: crystallographic data, hydrogen-bond parameters for the studied compound at different temperatures, structural and optical parameters for selected A_2_PbBr_4_ perovskites (PDF)
Video: Modulation movie of phase II (MPG)

## Supplementary Material

ic2c02206_si_001.pdf

ic2c02206_si_002.mpg
