# Supplementary material for: [Methylhydrazinium]2PbCl4, a Two-Dimensional Perovskite with Polar and Modulated Phases
Source: Inorg Chem. 2022 Sep 21;61(39):15520–31. doi: 10.1021/acs.inorgchem.2c02206 (PMC9533301; doi:10.1021/acs.inorgchem.2c02206)
Supplement: Supplementary file 1 — ic2c02206_si_001.pdf [file ic2c02206_si_001.pdf]

Supporting information for

[Methylhydrazinium]$_2$PbCl$_4$, a Two-dimensional Perovskite with Polar and Modulated Phases

Katarzyna Fedoruk[a], Dawid Drozdowski[b], Mirosław Mączka*[b], Jan K. Zaręba[c], Dagmara Stefańska[b], Anna Gągor[b], and Adam Sieradzki*[a]

[a]*Department of Experimental Physics, Wrocław University of Science and Technology, Wybrzeże Wyspiańskiego 27, 50-370 Wrocław, Poland*

[b]*Institute of Low Temperature and Structure Research, Polish Academy of Sciences, ul. Okólna 2, 50-422 Wrocław, Poland*

[c]*Advanced Materials Engineering and Modeling Group, Wrocław University of Science and Technology, Wybrzeże Wyspiańskiego 27, 50-370, Wrocław, Poland*

e-mails: m. maczka@intibs.pl; adam.sieradzki@pwr.edu.pl

Table S1. Experimental and refinement details of MHy_2PbCl_4 (M_r = 443.16)

	Phase I	Phase II	Phase III
Crystal data			
Crystal system, space or superspace group	Orthorhombic, *Pmmn*	Orthorhombic, *Pmmn*(00*γ*)*s*00†	Monoclinic, $P2_1$
Temperature (K)	350	295	120
a, *b*, *c* (Å)	5.7902 (1), 17.5814 (6), 5.8657 (1)	5.7800 (1), 17.3224 (3), 5.8731 (1)	11.6588 (5), 17.0423 (6), 12.7453 (6)
α, *β*, *γ* (°)	90, 90, 90	90, 90, 90	90, 114.16 (1), 90
V (Å^3)	597.13 (3)	588.04 (2)	2310.65 (18)
Z	2	2	8
μ (mm^{-1})	14.98	15.21	15.48
Crystal size (mm)	0.22 × 0.08 × 0.06	0.16 × 0.13 × 0.05	0.16 × 0.13 × 0.05
Data collection			
T_{min}, T_{max}	0.407, 1.000	0.393, 1.000	0.745, 1.000
No. of measured, independent and observed reflections	6680, 699, 682 [$I > 2\sigma(I)$]	71262, 2386, 1517 [$I > 4\sigma(I)$]	13396, 13396, 10108 [$I > 2\sigma(I)$]
R_{int}	0.032	0.032	
(sin $\theta/\lambda)_{max}$ (Å^{-1})	0.625	0.624	0.610
Refinement			
$R[F^2 > 2\sigma(F^2)]$, $wR(F^2)$, S (all)	0.018, 0.045, 1.13	0.029, 0.062, 1.88	0.057, 0.169, 1.02
$R[F^2 > 2\sigma(F^2)]$, $wR(F^2)$ (main)		0.022, 0.032	
$R[F^2 > 2\sigma(F^2)]$, $wR(F^2)$ (satellites)		0.086, 0.117	
No. of reflections	699	2386	13396
No. of parameters	33	52	318
No. of restraints	0	0	186
$\Delta\rho_{max}$, $\Delta\rho_{min}$ (e Å^{-3})	1.13, -0.69	0.65, -1.06	3.45, -2.70
Absolute structure parameter	–	–	0.48 (4)

Table S2. Selected hydrogen-bond parameters in **I**.

D—H···*A*	*D*—H (Å)	H···*A* (Å)	*D*···*A* (Å)	*D*—H···*A* (°)
N2—H2A···Cl3^{i}	0.91	2.68	3.421 (6)	138.9
N1—H1D···Cl1^{ii}	0.89	2.56	3.429 (14)	167.3
N1—H1E···Cl3^{ii}	0.89	2.65	3.168 (12)	118.0

Symmetry codes: (i) *x*-1, *y*, *z*-1; (ii) *x*, *y*, *z*-1.

Table S3. Selected hydrogen-bond parameters in **III**.

D—H···*A*	*D*—H (Å)	H···*A* (Å)	*D*···*A* (Å)	*D*—H···*A* (°)
N1—H1D···Cl16^{iv}	0.91	2.57	3.31 (3)	138.9
N1—H1D···Cl9	0.91	2.65	3.23 (3)	122.1
N2—H2A···Cl10^{vi}	0.85	2.73	3.47 (3)	145.6
N2—H2B···Cl15^{iv}	0.80	2.62	3.36 (3)	153.4
N3—H3D···Cl11	0.91	2.37	3.18 (4)	147.9
N3—H3E···Cl15	0.91	2.25	3.12 (3)	161.1
N4—H4D···Cl14^{viii}	0.89	2.37	3.25 (3)	170.7
N5—H5A···Cl10	0.91	2.37	3.23 (3)	157.6
N5—H5B···Cl14^{iv}	0.91	2.30	3.14 (3)	154.1
N7—H7B···Cl13	0.91	2.61	3.37 (3)	141.3
N7—H7B···Cl14	0.91	2.70	3.23 (3)	118.8
N8—H8A···Cl14	0.91	2.80	3.39 (3)	123.7
N8—H8A···Cl15^{i}	0.91	2.69	3.41 (3)	137.4
N8—H8B···Cl10	0.92	2.55	3.35 (4)	146.1
N9—H9A···Cl10^{vii}	0.91	2.38	3.17 (4)	145.5
N9—H9B···Cl7	0.91	2.30	3.18 (4)	160.9
N10—H10A···Cl6^{vii}	0.90	2.54	3.36 (4)	152.1
N10—H10B···Cl8	0.90	2.66	3.47 (4)	149.8
N11—H11B···Cl4	0.91	2.44	3.27 (4)	151.2
N12—H12A···Cl2^{v}	0.90	2.61	3.31 (4)	135.5
N12—H12B···Cl7	0.90	2.40	3.25 (4)	159.2
N13—H13A···Cl8	0.91	2.50	3.34 (4)	153.4
N14—H14A···Cl7^{ii}	0.91	2.49	3.22 (4)	137.3
N14—H14B···Cl2^{iii}	0.91	2.34	3.22 (4)	164.2
N15—H15A···Cl2^{iii}	0.91	2.17	3.06 (4)	168.3
N15—H15B···Cl15^{x}	0.91	2.54	3.30 (4)	141.4
N16—H16A···Cl3^{ii}	0.90	2.38	3.27 (4)	171.6
N16—H16B···Cl4^{iii}	0.90	2.77	3.60 (4)	154.4
N1—H1E···N14^{vii}	0.91	2.17	3.00 (5)	149.8
N4—H4E···N16^{ix}	0.89	2.22	3.09 (5)	163.0

N7—H7A···N12^{ii}	0.91	1.97	2.87 (5)	172.2
N9—H9A···N8^{vii}	0.91	2.62	3.16 (5)	118.8
N11—H11A···N6^{v}	0.91	1.91	2.81 (5)	169.5
N13—H13B···N4^{ii}	0.91	1.97	2.87 (5)	168.4
N15—H15B···N2^{ii}	0.91	2.48	3.06 (5)	122.2

Symmetry codes: (i) -*x*, *y*-1/2, -*z*; (ii) -*x*+1, *y*-1/2, -*z*+1; (iii) *x*-1, *y*, *z*; (iv) *x*+1, *y*, *z*; (v) -*x*+2, *y*+1/2, -*z*+1; (vi) -*x*+1, *y*+1/2, -*z*; (vii) -*x*+1, *y*+1/2, -*z*+1; (viii) -*x*, *y*+1/2, -*z*; (ix) -*x*, *y*+1/2, -*z*+1; (x) -*x*, *y*-1/2, -*z*+1.

Table S4. Comparison of structural parameters for A_2PbCl_4 perovskites with short separation between layers.

Compound	**Interlayer distance at RT[a] (Å)**	Reference
MHy_2PbCl_4[b]	**8.661**	This work
$DMEDAPbCl_4$	**9.090**	([1])
$AMPyrPbCl_4$	**9.209**	([2])
$DMPDAPbCl_4$	**9.864**	([3])
TzH_2PbCl_4	**10.796**	([4])
$PDAPbCl_4$	**11.297**	([5])
$2MEPTPbCl_4$	**11.887**	([6])
BA_2PbCl_4	**13.993**	([7])

[a] – Interlayer distance is calculated as a distance between Pb atoms. If the out-of-plane octahedral distortion occurs, a plane through the averaged Pb positions is used as a reference, RT denotes to room temperature.

MHy – methylhydrazinium, DMEN – *N,N*-dimethylethylenediamine, AMPyr – 2-aminomethylpyridinium, DMEDA – *N,N*-dimethyl-1,3-diaminopropane, TzH – 1,2,4-triazolium, PDA – pentamethylenediammonium, 2MEPT – 2-methyl-1,5-diaminopentane, BA – butylammonium.

Table S5. Raman wavenumbers (in cm^{-1}, λ_{exc}=830 nm) for MHy_2PbCl_4 single crystals measured for x(yy+yz)x and x(zz+yz)x polarization at 365, 225 and 80 K together with the proposed assignment.[a]

x(yy+yz)x A$_1$(TO)+ B$_2$(TO)			x(zz+zy)x A$_1$(TO)+ B$_2$(TO)			assignment
365 K	225 K	80 K	365 K	225 K	80 K	
	231w	223w+236w			240vw	L(MHy^+)+T'(MHy^+)+Pb-Cl stretch
186sh	182w	192w+175w	164m,vb	182m,b	187m	L(MHy^+)+T'(MHy^+)+Pb-Cl stretch
		154w+143w			145vw	L(MHy^+)+T'(MHy^+)+Pb-Cl stretch
		127w+132w			133vw+123vw	Pb-Cl bend
105vs,vb	112vs,b	120vs+ 106s	90vs,vb	113sh+ 92vs	108s+ 97s	Pb-Cl bend
		96w+93w				Pb-Cl bend
73s,vb	83s,b	88w+83s+80w+ 76w	69vs,b	77sh	89s	Pb-Cl bend
		69m+63w+57w				Pb-Cl bend
	55m	50s	54vs,b	58sh	76m+67s	Pb-Cl bend
		35s+39w			52w+48w	Pb-Cl bend
26m	28m	29w	23m	25s	28s	L($PbCl_6$)
	16m	24w		16s		L($PbCl_6$)

[a]Key: vs, very strong; s, strong; m, medium; w, weak; vw, very weak; sh, shoulder; b, broad.

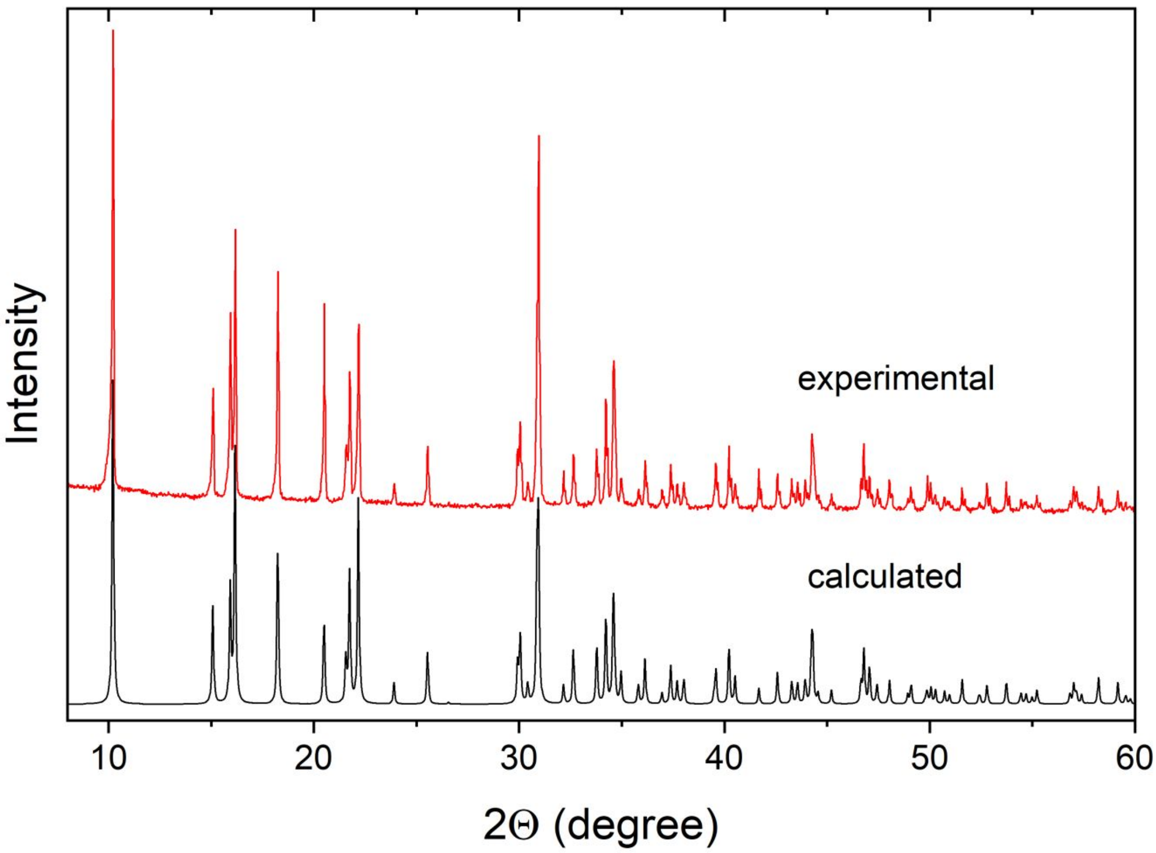

Figure S1. Powder XRD pattern for MHy_2PbCl_4 together with the calculated one based on the single crystal structure at room temperature.

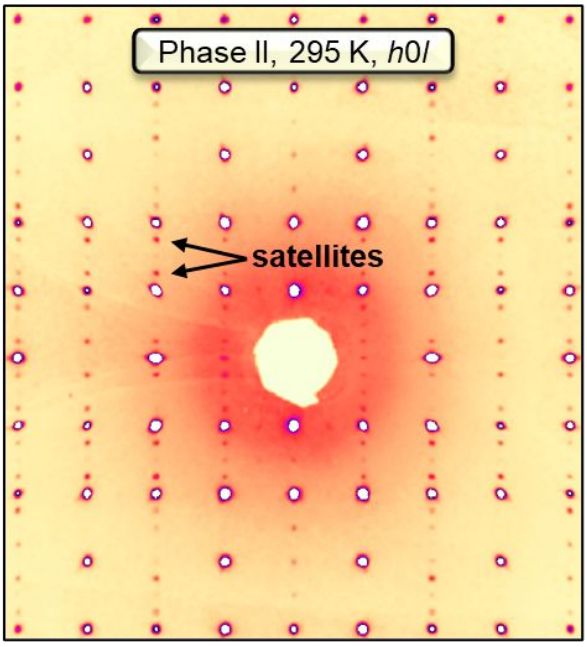

Figure S2. Reciprocal space reconstruction of the *h0l* layer in phase **II** with first-order satellite reflections spotted along *c**, pointing out to the modulated structure with modulation vector **q** ≅ 0.25**c***.

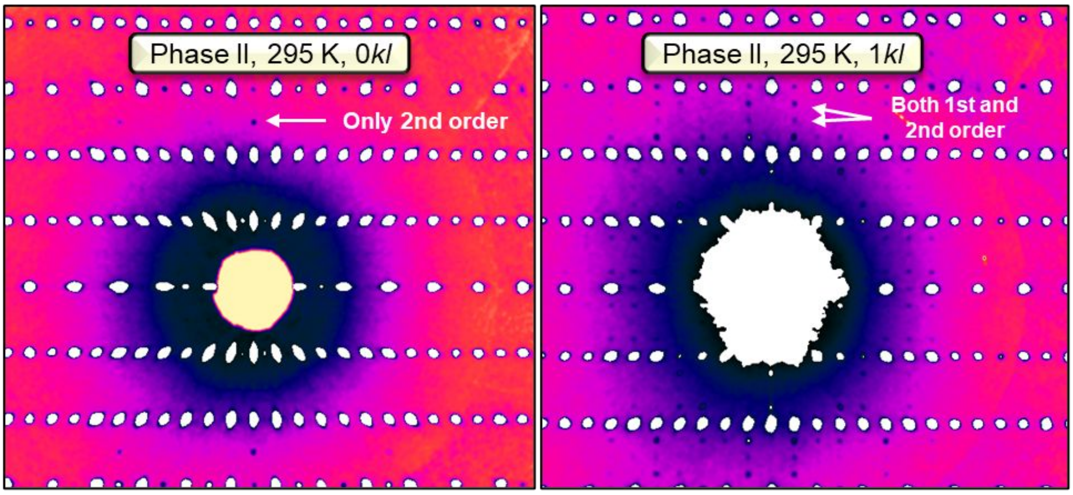

Figure S3. Reciprocal space reconstruction of the 0*kl* and 1*kl* layers in phase **II**. In order to apply the systematic extinctions rule for satellite reflections, four integers *hklm* (*h**a*** + k**b*** + l**c*** + m**q***) are required to index all peaks. For *0klm* layer, the only visible satellite reflections are for *m* = 2*n*. According to the *International Tables for Crystallography, Vol. C*, chapter

"Incommensurate and commensurate modulated structures",[8] this rule applies to an intrinsic shift *s* along the [100] direction. Therefore, the (3+1)-dimensional superspace group is $Pmmn(00\gamma)s00$.

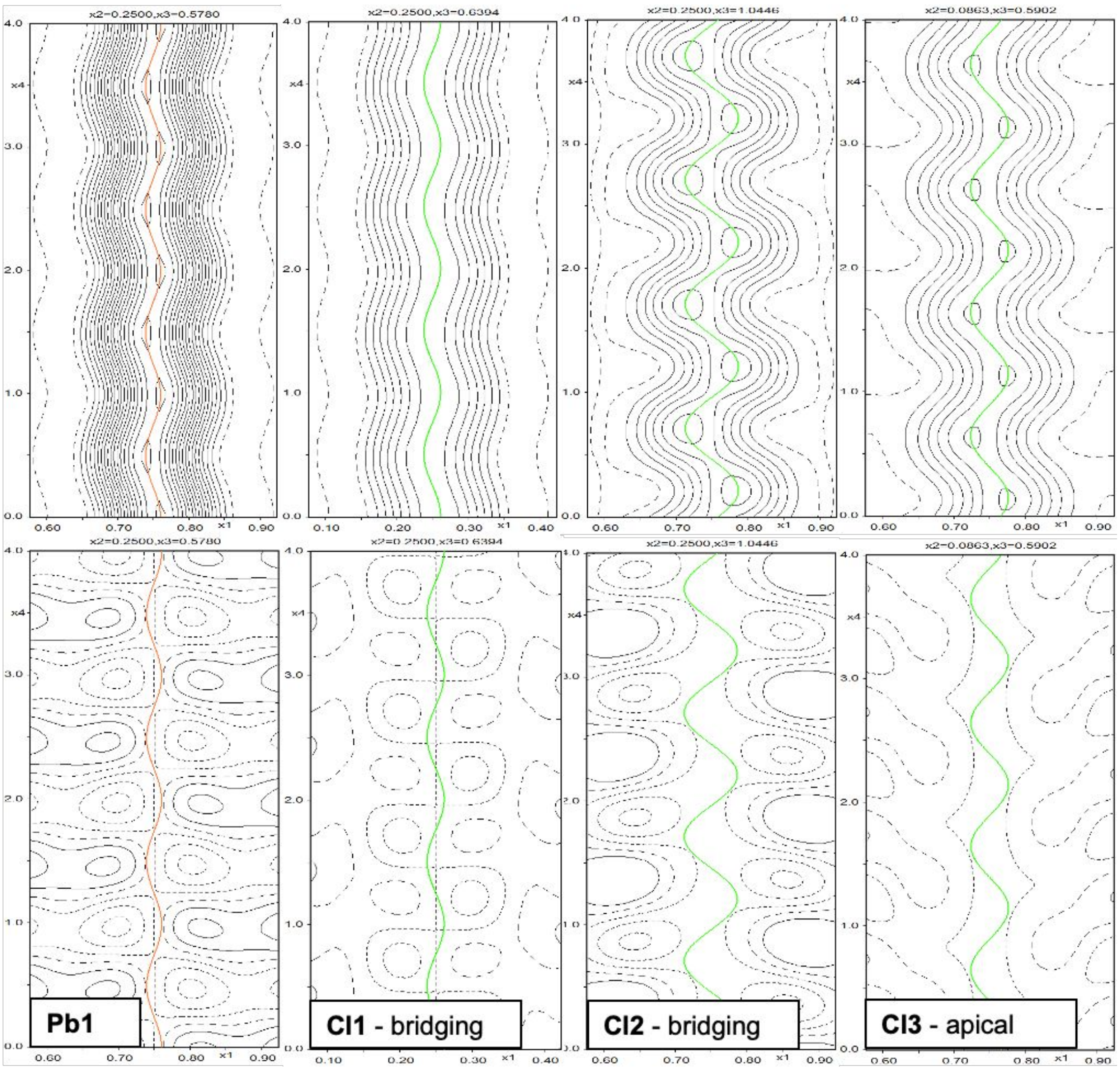

Figure S4. The x1-x4 sections through Pb (orange) and three inequivalent Cl atoms (green) in **II**. The contour step is 20 e Å^{-3} for Pb and 5 e Å^{-3} for Cl. The dashed lines represent negative densities. At the bottom the difference Fourier maps are presented. The contour step is 0.5 e Å^{-3} for Pb and 0.3 e Å^{-3} for Cl.

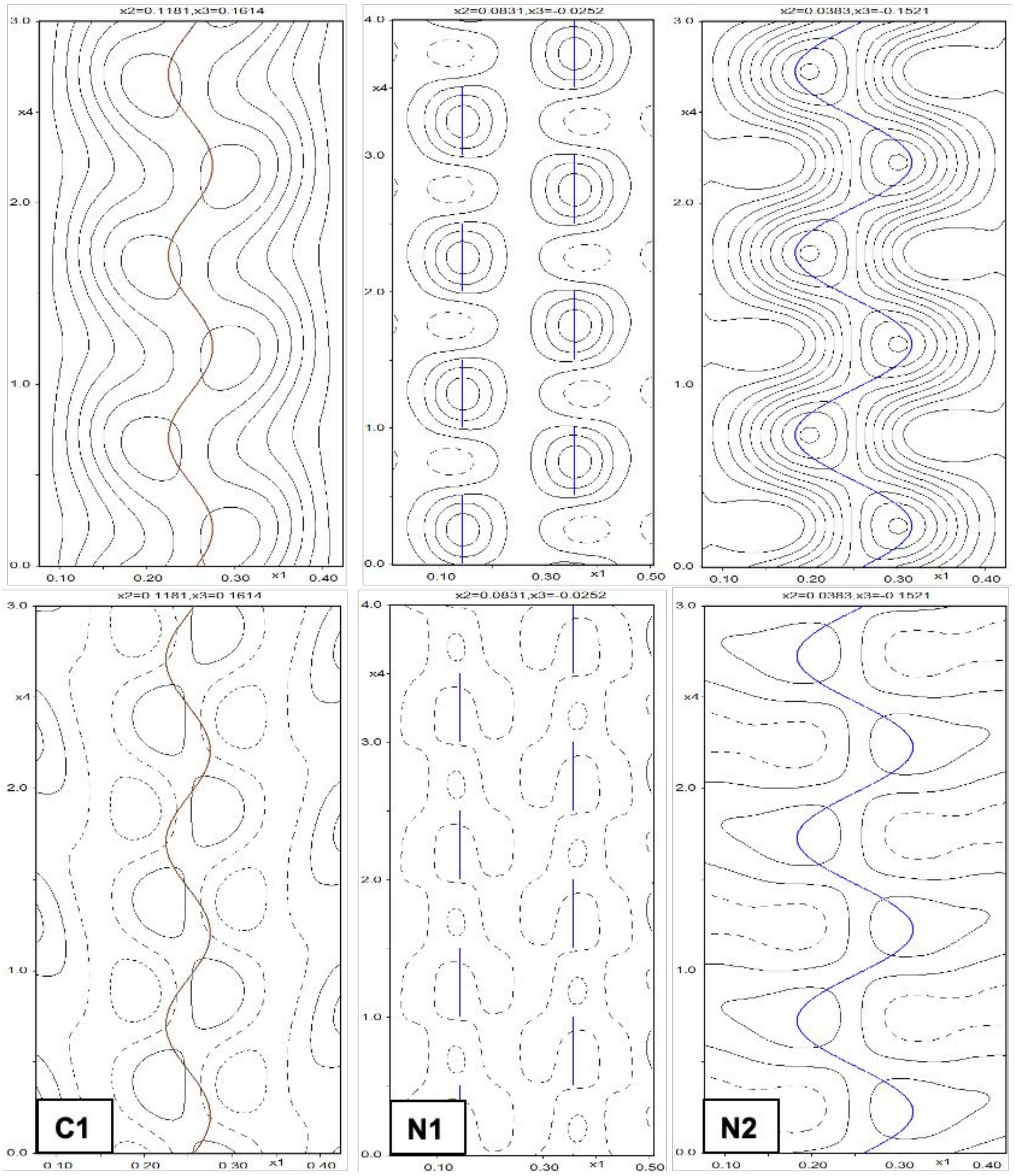

Figure S5. The x1-x4 sections through C (brown) N atoms (blue) in **II**. The contour step is 1 e Å^{-3} for all atoms. The dashed lines represent negative densities. At the bottom the difference Fourier maps are presented. The contour step is 0.2 e Å^{-3} for all atoms.

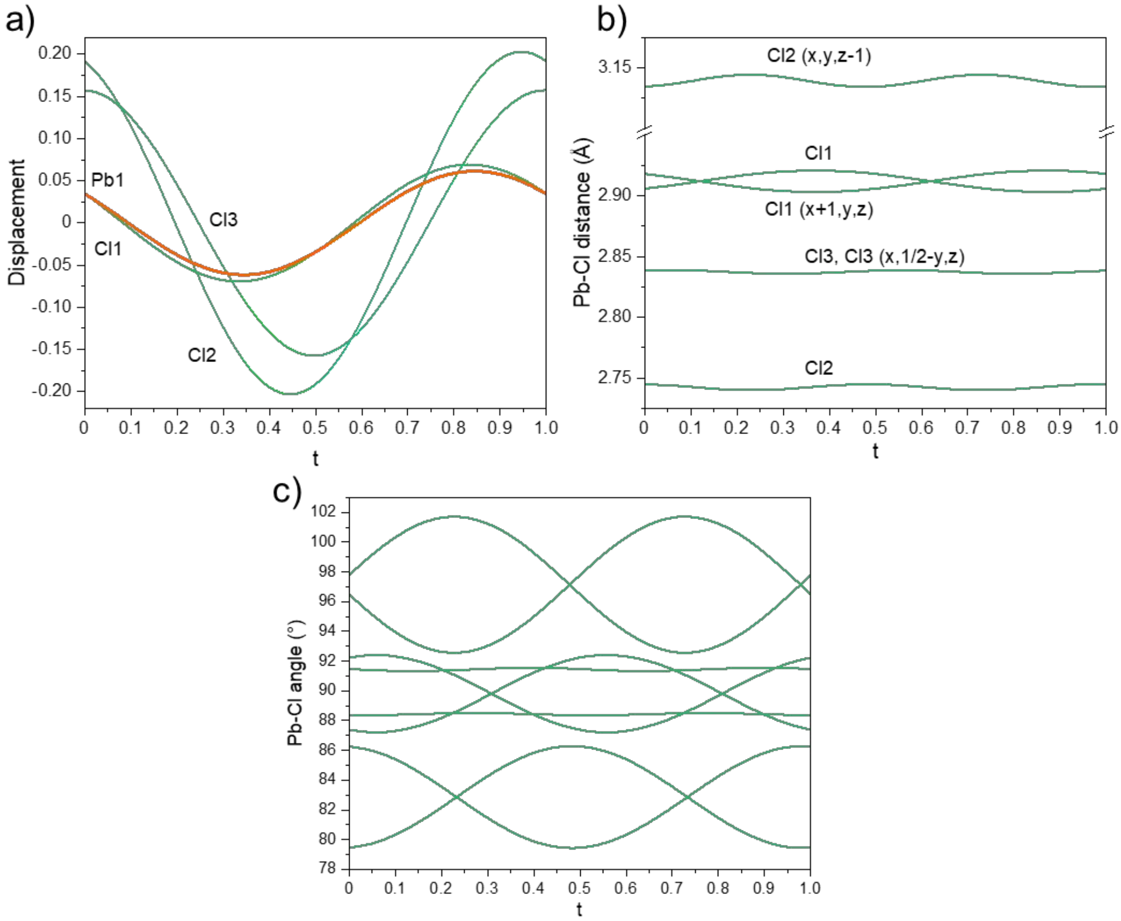

Figure S6. (a) Displacement of independent Pb and Cl atoms of MHy_2PbCl_4 in **II** along the *a* direction as function of the phase of the modulation *t.* (b) Pb-Cl distances with grey dashed lines corresponding to distances in **I**. (c) Cl-Pb-Cl angles.

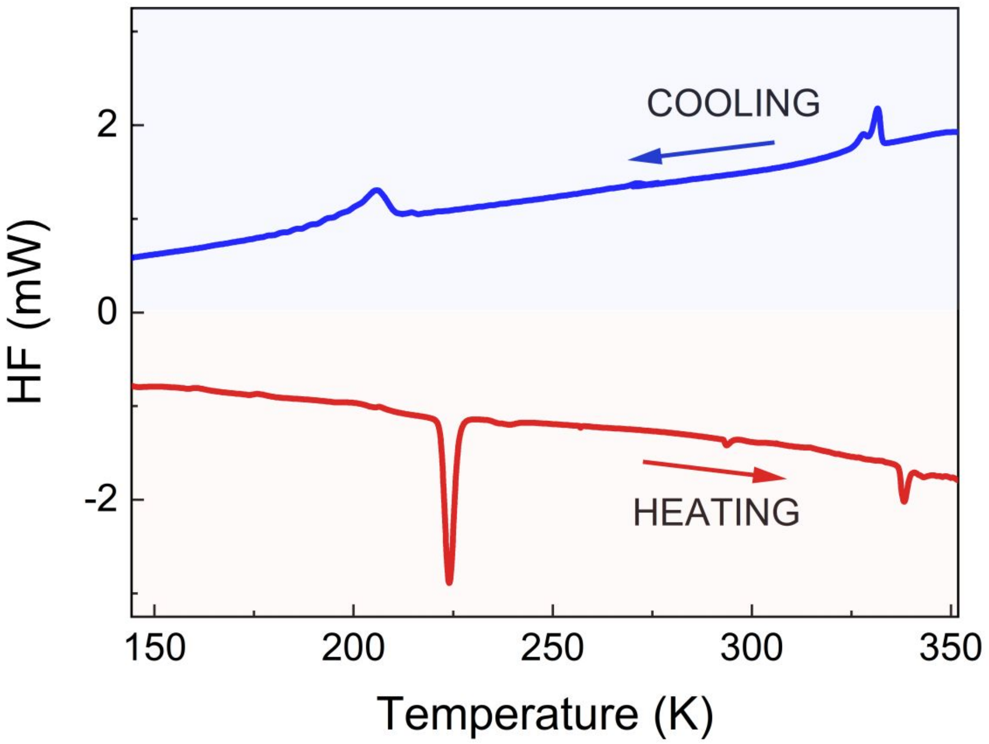

Figure S7. DSC trace of MHy_2PbCl_4.

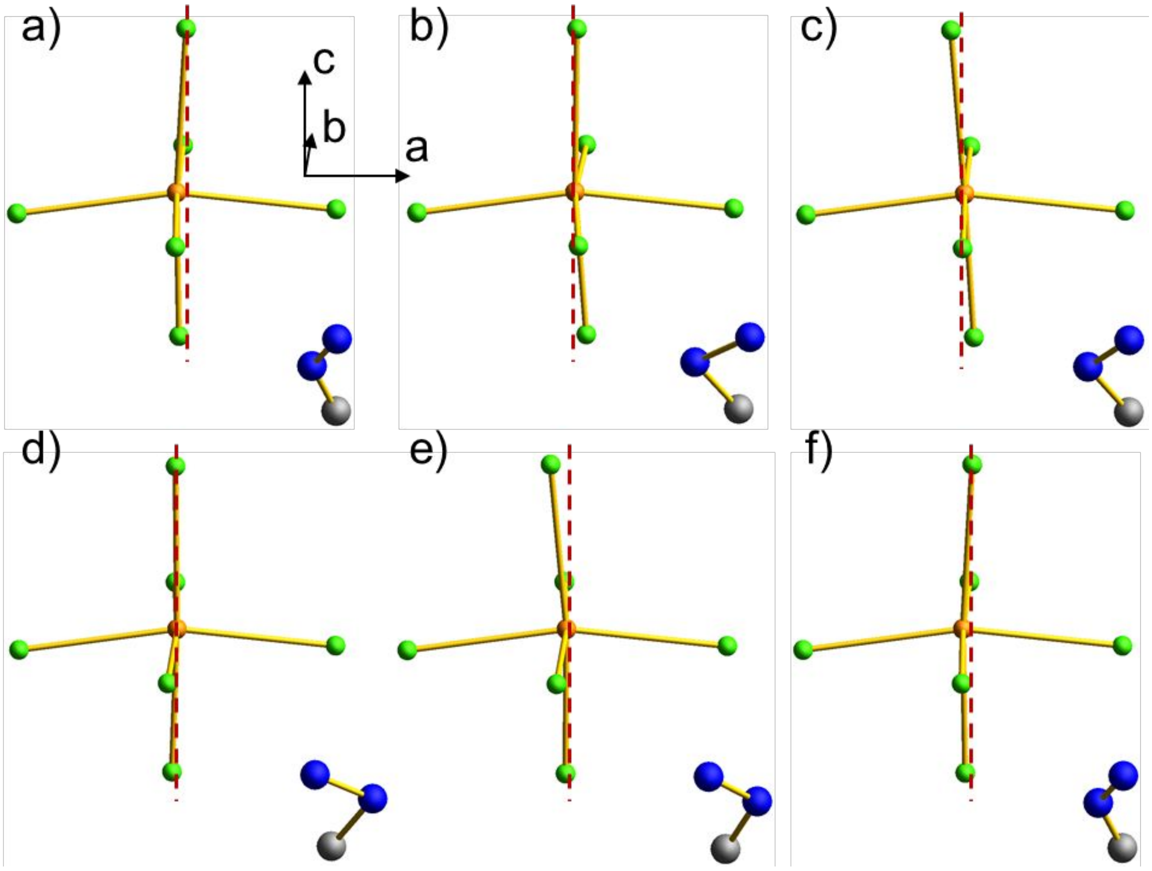

Figure S8. Snapshots of the modulation in phase **II** at 295 K, showing single $PbCl_6$ octahedra and MHy^+ cation for six distinct *t* values: a) 0, b) 0.2, c) 0.4, d) 0.6, e) 0.8 and f) 1.0. Red dashed lines pass through x = 0.25.

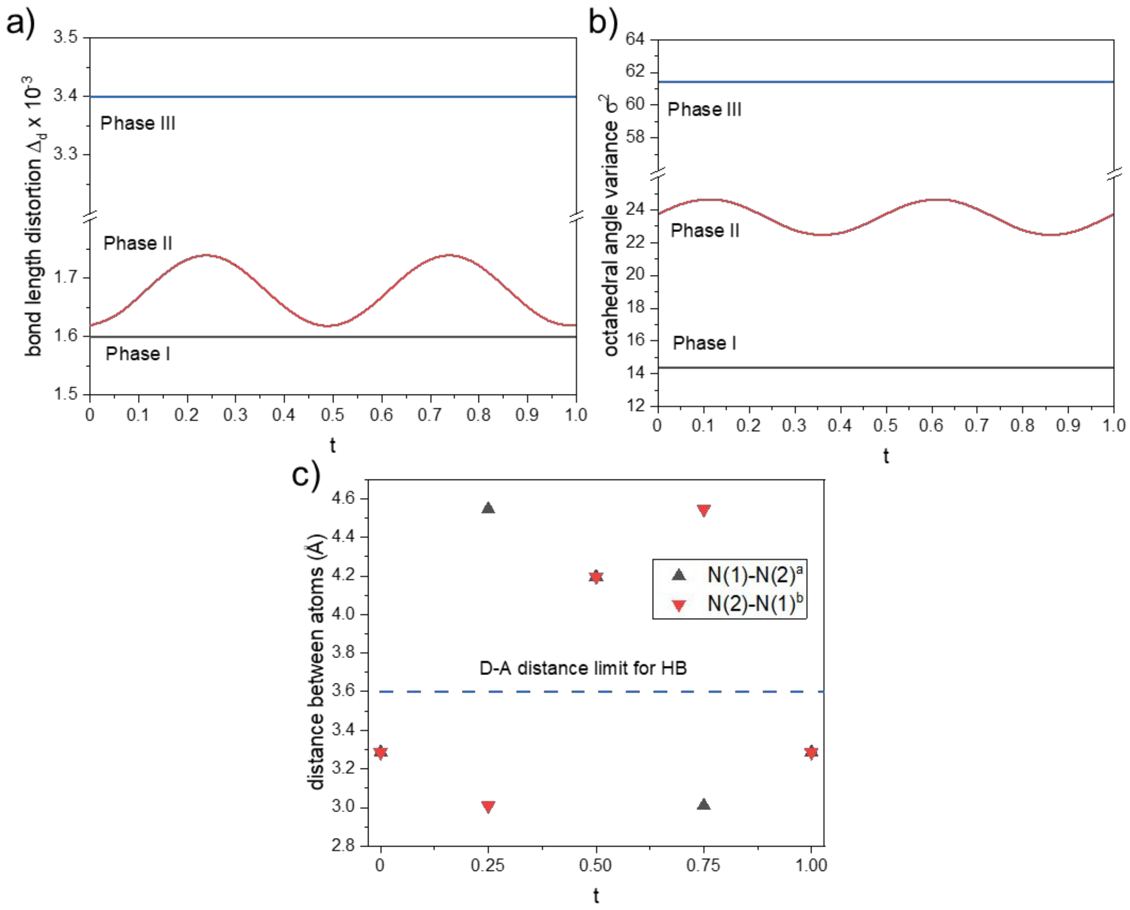

Figure S9. (a, b) Octahedral (a) bond length distortion and (b) angle variance for modulated phase **II** as the function of the phase of the modulation *t*. Grey (blue) straight lines correspond to values in non-modulated phase **I** (**III**). The least distorted octahedra, in terms of the bond length distortion (Δ_d) values are for $t = 0$ and 0.50, where Δ_d value is almost equal to the one recorder in phase **I** (1.6 x 10^{-3}), while for $t = 0.25$ and 0.75 Δ_d is the largest (1.74 x 10^{-3}). (c) Distance between opposite N atoms from neighboring MHy molecules, where [a] denotes 1/2-x,-y,-z and [b] denotes 1-x,-y,-z symmetry operation. Red dashed line corresponds to the donor-acceptor distance limit for hydrogen bonding, according to the Jeffrey criterion.[9]

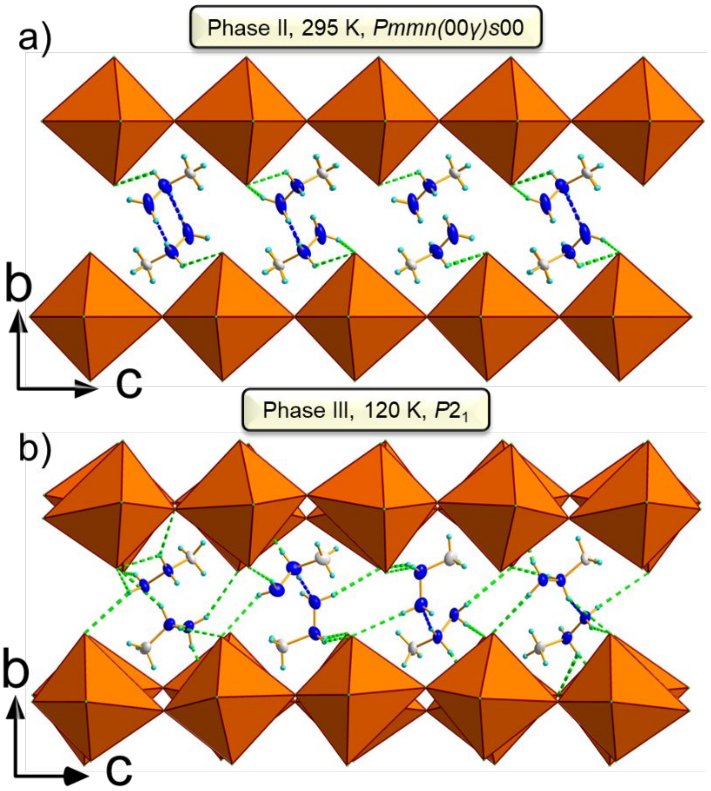

Figure S10. Comparison of hydrogen bonding configuration in (a) phase **II** and (b) **III**. Green and blue dashed lines correspond to N-H···Cl and N-H···N HBs, respectively.

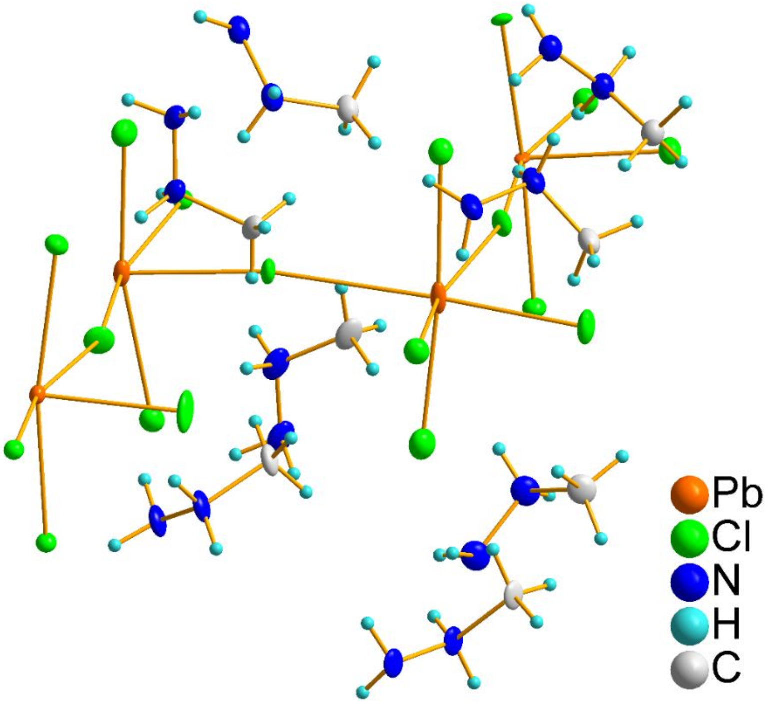

Figure S11. The asymmetric unit of **III**, consisting of four Pb and eight Cl inequivalent atoms, and eight inequivalent, ordered MHy^+ cations.

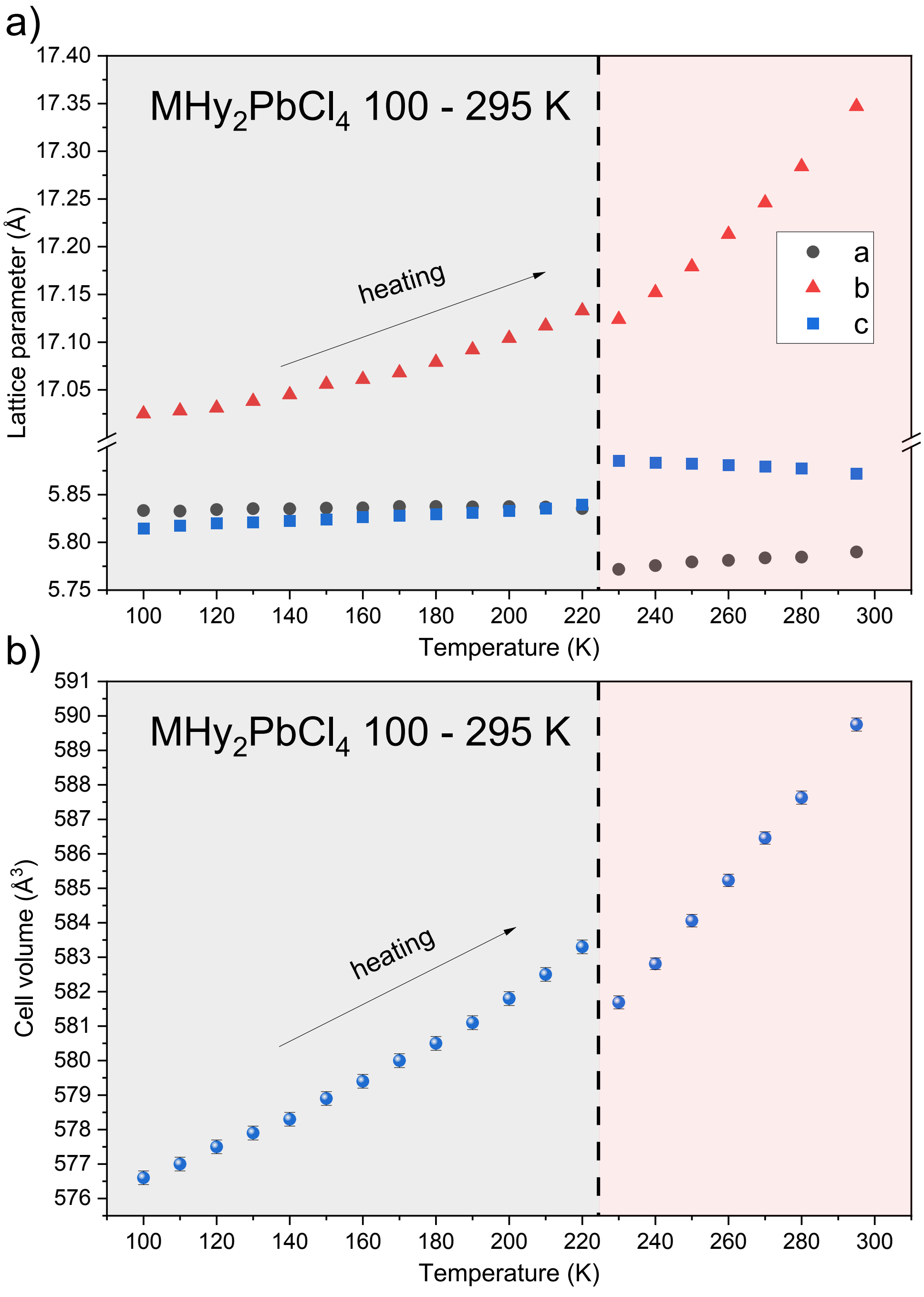

Figure S12. Geometric parameters of MHy_2PbCl_4 as function of the temperature in a heating run. (a) Lattice cell parameters, (b) unit cell volume. Light-grey (light-red) field correspond to phase **III** (**II**). Black dashed-line denotes to the T_c obtained from DSC measurements.

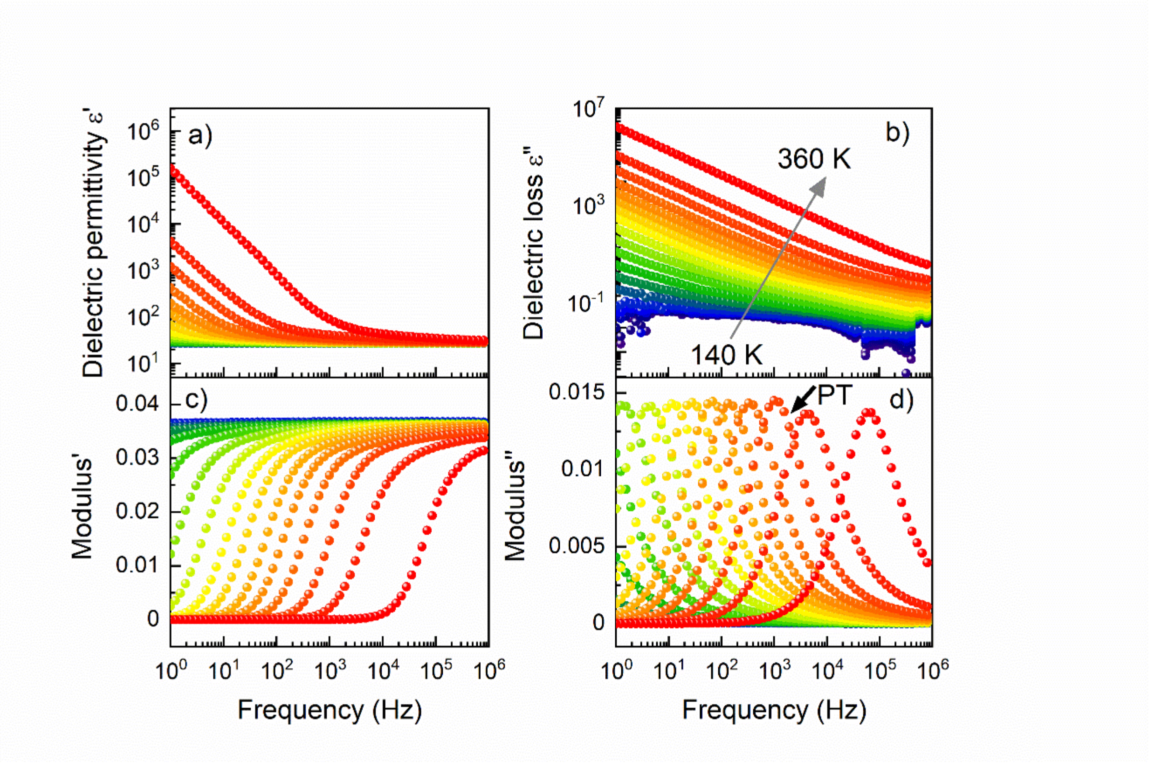

Figure S13. Frequency dependence of the a) dielectric permittivity, b) dielectric loss, c) real M′, and d) imaginary M″.

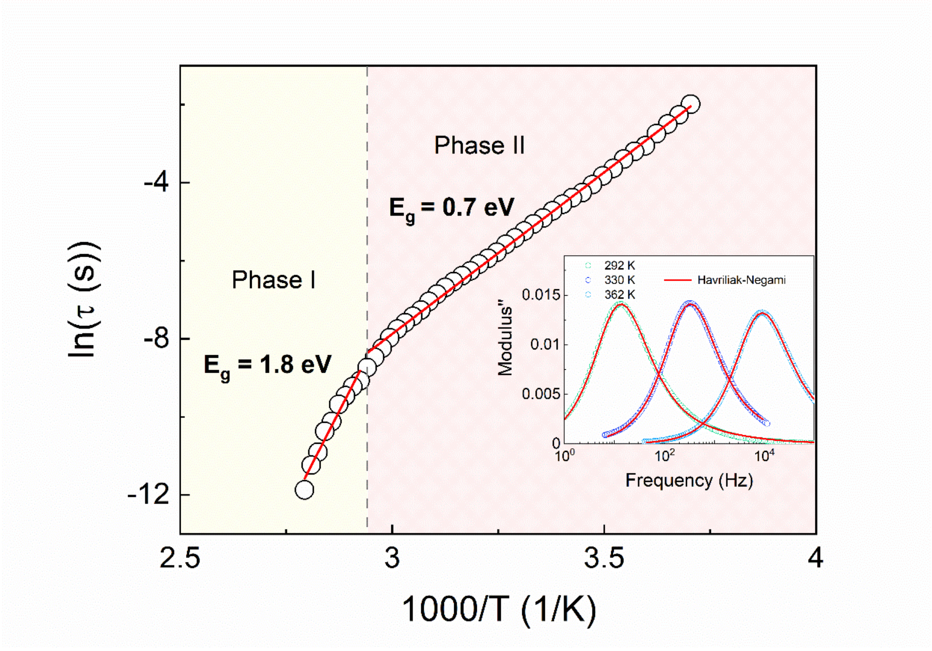

Figure S14. The estimated relaxation times of MHy^+ motion as a function of inverse temperature. Insert shows examples of fits to modulus values as a function of frequency. Dash line corresponds to the structural phase transition temperature.

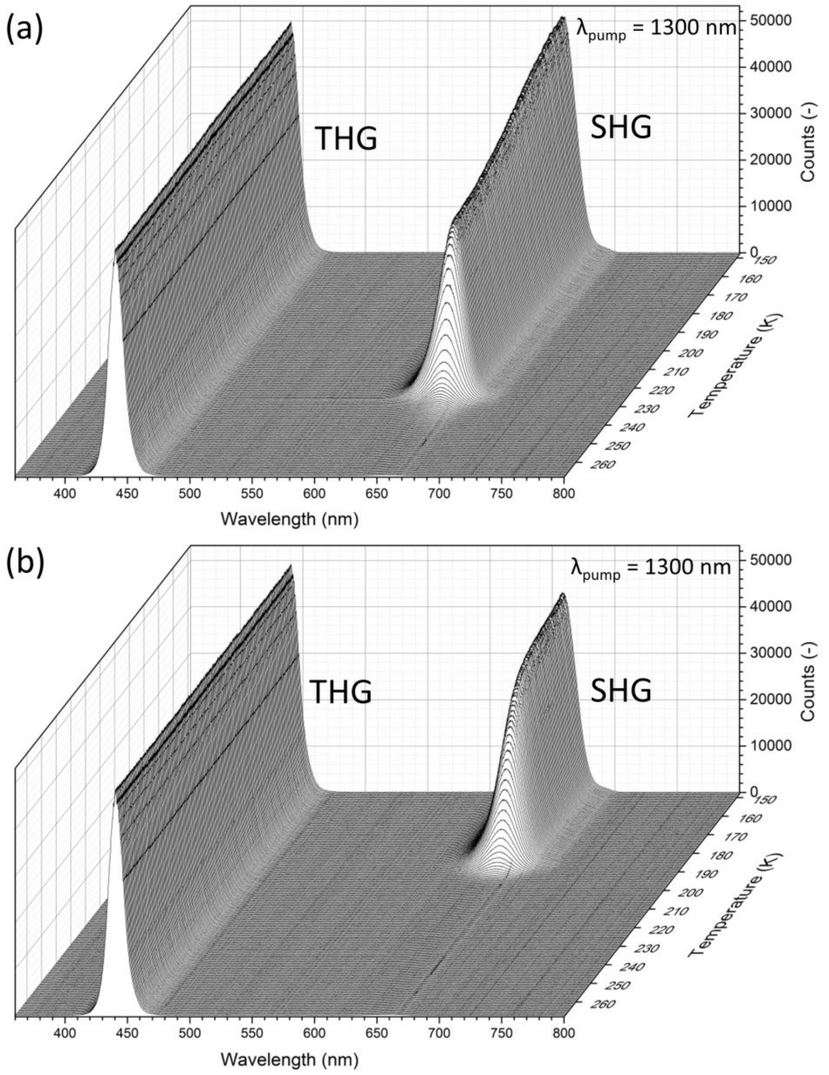

Figure S15. Spectra obtained upon irradiation with 1300 nm femtosecond laser pulses of MHy_2PbCl_4 during (a) heating run from 148 K to 268 K and (b) cooling run from 268 K to 148 K. It is clear that low temperature phase **III** emits SHG hence is noncentrosymmetric, whereas phase **II** is not.

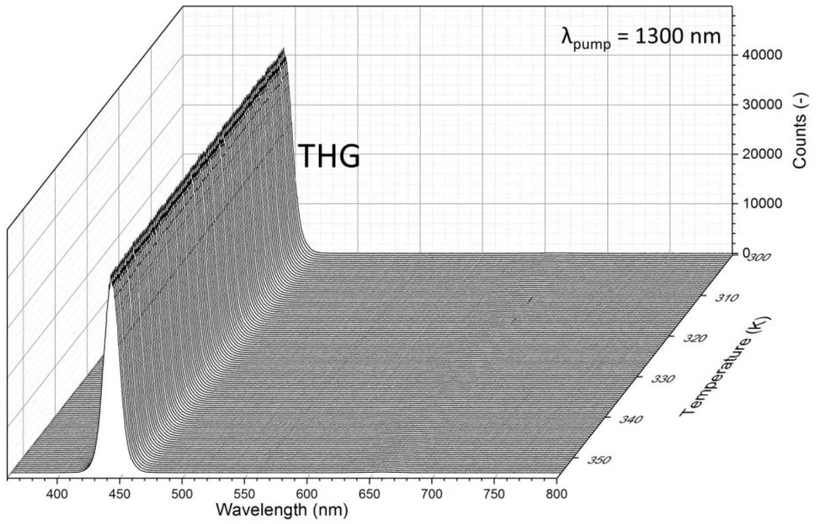

Figure S16. Spectra obtained upon irradiation with 1300 nm femtosecond laser pulses of MHy_2PbCl_4 during heating from 300 K to 355 K. These data show that neither phase **I** nor phase **II** of MHy_2PbCl_4 is noncentrosymmetric.

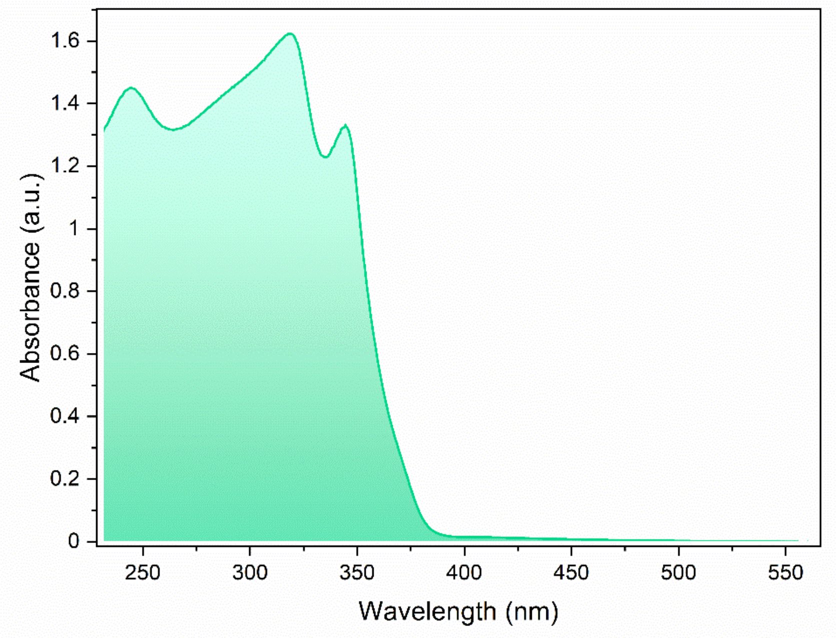

Figure S17. Diffuse reflectance spectra of MHy_2PbCl_4 recorded at room temperature.

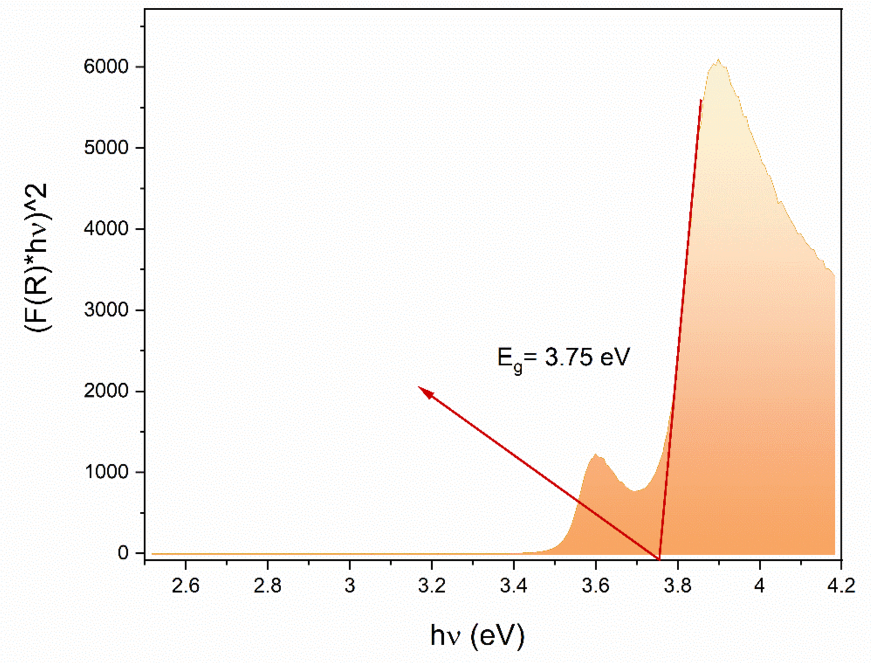

Figure S18. Energy band gap of investigated MHy_2PbCl_4 determined using Kubelka-Munk notation.

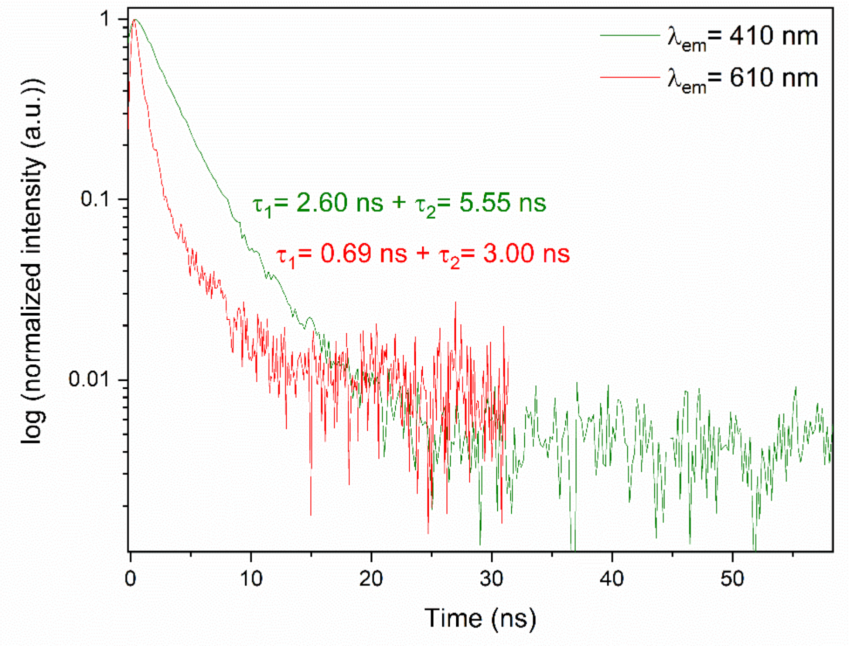

Figure S19. Emission decay curves of MHy_2PbCl_4 monitored at λ= 410 nm and λ= 610 nm at 80 K.

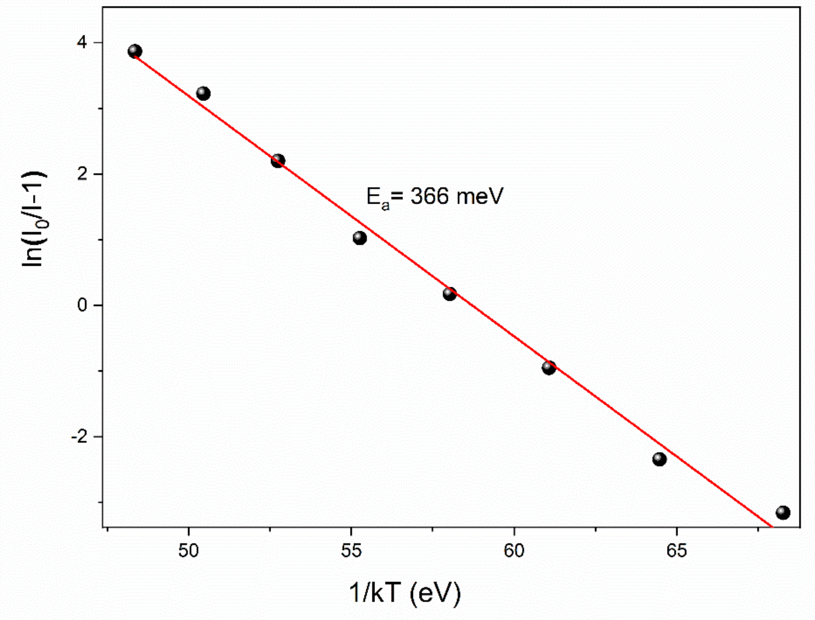

Figure S20. Energy activation for thermal quenching of investigated compound.

REFERENCES

(1) Rong, L.-Y.; Hu, C.; Xu, Z.-N.; Wang, G.-E.; Guo, G.-C. 2D Perovskite Hybrid with Both Semiconductive and Yellow Light Emission Properties. *Inorg. Chem. Commun.* **2019**, *102*, 90–94. https://doi.org/10.1016/j.inoche.2019.01.042.

(2) Lermer, C.; Senocrate, A.; Moudrakovski, I.; Seewald, T.; Hatz, A.-K.; Mayer, P.; Pielnhofer, F.; Jaser, J. A.; Schmidt-Mende, L.; Maier, J.; Lotsch, B. V. Completing the Picture of 2-(Aminomethylpyridinium) Lead Hybrid Perovskites: Insights into Structure, Conductivity Behavior, and Optical Properties. *Chem. Mater.* **2018**, *30* (18), 6289–6297. https://doi.org/10.1021/acs.chemmater.8b01840.

(3) Jing, C.; Wang, J.; Zhao, H.; Chu, W.; Yuan, Y.; Wang, Z.; Han, M.; Xu, T.; Zhao, J.; Lei, X. Improving Broadband White-Light Emission Performances of 2D Perovskites by Subtly Regulating Organic Cations. *Chem. – A Eur. J.* **2020**, *26* (45), 10307–10313. https://doi.org/10.1002/chem.202001178.

(4) Guo, Y.-Y.; Yang, L.-J.; McNulty, J. A.; Slawin, A. M. Z.; Lightfoot, P. Structural Variations in (001)-Oriented Layered Lead Halide Perovskites, Templated by 1,2,4-Triazolium. *Dalt. Trans.* **2020**, *49* (47), 17274–17280. https://doi.org/10.1039/D0DT02936J.

(5) Yang, W.; Xiao, X.; He, H.; Tong, G.; Hu, J.; Xiao, X.; Chen, J.; Li, M.; He, Y.

Intermolecular Hydrogen-Bonding Correlated Structure Distortion and Broadband White-Light Emission in 5-Ammonium Valeric Acid Templated Lead Chloride Perovskites. *Cryst. Growth Des.* **2021**, *21* (10), 5731–5739. https://doi.org/10.1021/acs.cgd.1c00566.

(6) Wang, S.; Yao, Y.; Wu, Z.; Peng, Y.; Li, L.; Luo, J. Realization of “Warm” White Light *via* Halide Substitution in Polar Two-Dimensional Hybrid Perovskites $(2meptH_2)PbCl_xBr_{4-x}$. *J. Mater. Chem. C* **2018**, *6* (45), 12267–12272. https://doi.org/10.1039/C8TC03167C.

(7) Tu, Q.; Spanopoulos, I.; Vasileiadou, E. S.; Li, X.; Kanatzidis, M. G.; Shekhawat, G. S.; Dravid, V. P. Exploring the Factors Affecting the Mechanical Properties of 2D Hybrid Organic–Inorganic Perovskites. *ACS Appl. Mater. Interfaces* **2020**, *12* (18), 20440–20447. https://doi.org/10.1021/acsami.0c02313.

(8) Janssen, T.; Janner, A.; Looijenga-Vos, A.; de Wolff, P. M. Incommensurate and Commensurate Modulated Structures. In *International Tables for Crystallography*; International Union of Crystallography: Chester, England, 2006; pp 907–955. https://doi.org/10.1107/97809553602060000624.

(9) George A. Jeffrey. An Introduction to Hydrogen Bonding. *Oxford Univ. Press* **1997**.
